# Morphine coordinates SST and PV interneurons in the prelimbic cortex to disinhibit pyramidal neurons and enhance reward

**DOI:** 10.1038/s41380-019-0480-7

**Published:** 2019-08-14

**Authors:** Changyou Jiang, Xueying Wang, Qiumin Le, Peipei Liu, Cao Liu, Zhilin Wang, Guanhong He, Ping Zheng, Feifei Wang, Lan Ma

**Affiliations:** Department of Neurology, State Key Laboratory of Medical Neurobiology and MOE Frontiers Center for Brain Science, School of Basic Medical Sciences, Institutes of Brain Science and Zhongshan Hospital, Fudan University, 200032 Shanghai, China

**Keywords:** Neuroscience, Addiction

## Abstract

Opioids, such as morphine, are clinic analgesics which induce euphoria. Morphine exposure modifies the excitability and functional interactions between neurons, while the underlying cellular and molecular mechanisms, especially how morphine assembles heterogeneous interneurons (INs) in prelimbic cortex (PrL) to mediate disinhibition and reward, are not clear. Using approaches of optogenetics, electrophysiology, and cell type-specific RNA-seq, we show that morphine attenuates the inhibitory synaptic transmission from parvalbumin^+^ (PV)-INs onto pyramidal neurons in PrL via μ-opioid receptor (MOR) in PV-INs. Meanwhile, morphine enhances the inhibitory inputs from somatostatin^+^ (SST)-INs onto PV-INs, and thus disinhibits pyramidal neurons via δ-opioid receptor (DOR)-dependent Rac1 upregulation in SST-INs. We show that MOR in PV-INs is required for morphine-induced behavioral sensitization, while DOR as well as Rac1 activity in SST-INs is required for morphine-induced conditioned place preference and hyper-locomotion. These results reveal that SST- and PV-INs, functioning in PrL as a disinhibitory architecture, are coordinated by morphine via different opioid receptors to disinhibit pyramidal neurons and enhance reward.

## Introduction

Addictive drug-induced long-lasting modifications in the brain are associated with the neuronal plasticity in reward circuits, which depends on dopamine neurons in the ventral tegmental area (VTA) and their downstream targets, including nucleus accumbens (NAc), anterior cingulate cortex, medial prefrontal cortex (mPFC), etc. [1, 2]. mPFC responds diversely to reward-predictive cues and is critical for motivated behaviors [3]. Prelimbic subregion of mPFC (prelimbic cortex, PrL) has been implicated in regulation and innervation of addictive processes [4–9]. Both VTA and NAc are main recipients of the glutamatergic inputs from the PrL, indicating this nuclei is crucial for reward processing and the expression of drug-induced sensitization [3, 9, 10].

The information processing of cortical circuits depends on the functional interactions between the excitatory and inhibitory connectivity. Diverse types of GABAergic interneurons (INs) can receive, integrate, and encode information to stringently control the projective outputs [11]. Somatostatin (SST) and parvalbumin (PV) INs are two major subtypes of inhibitory neurons in PrL of rodent and human cortex, and they target distal dendritic and perisomatic regions of postsynaptic excitatory neurons, respectively, to exert their distinct inhibitory effects on excitatory neurons [12–15]. Functional connections between different subtypes of INs are observed and thus INs collaboratively regulate information integration in neural network [16–18]. The plasticity of PV-INs changes after fear conditioning and environment enrichment, and the dysfunction of PV-INs is found in schizophrenia mouse model [19, 20]. Abnormality of SST-INs in cortex is found in the Alzheimer’s mouse model [21]. These data indicate that the plasticity of distinct INs in cortical circuits participates in the process of cognition and psychiatric disorders.

Opioids, such as morphine, are clinical effective analgesics, but they also induce euphoria and adaptive changes of reward circuits [22]. Morphine acts through G-protein coupled opioid receptors to modulate presynaptic and postsynaptic ion channels [23–26] and disinhibit the inhibitory control to modulate pain and reward [27, 28]. Recent studies indicate that morphine exposure causes complex modifications of anatomical and functional connections in reward circuits, especially between excitatory neurons [29]. However, the effect and the biological basis of opioids on the connectivity among the excitatory and the inhibitory neurons in PrL, are not fully understood.

In this study, we combined morphological tracing, electrophysiology, and optogenetic methods, investigated morphine-induced alterations of inhibitory inputs from SST-INs and PV-INs onto local pyramidal neurons. Our results reveal a disinhibitory architecture consisting of SST- and PV-INs in the PrL, which is coordinated by morphine via different subtypes of opioid receptors to disinhibit pyramidal neurons, thus enhance morphine reward and related behaviors.

## Results

### Morphine attenuates the inhibitory transmission to pyramidal neurons from PV-INs, but not SST-INs in PrL via a MOR-dependent pathway

**Fig. 1:**
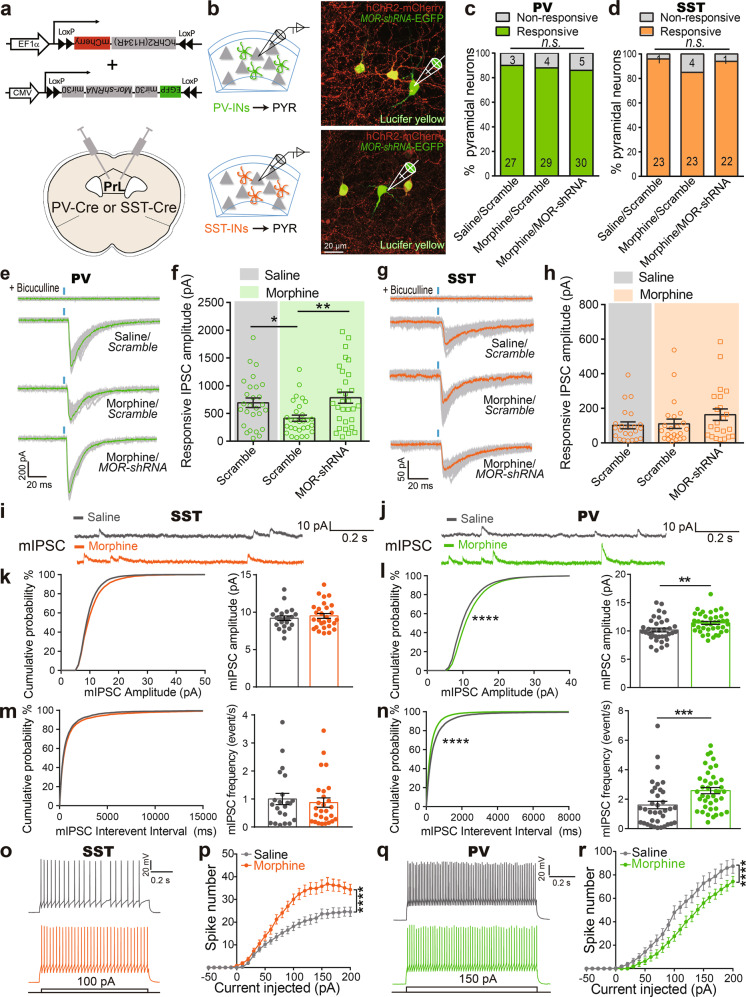
Morphine decreases the strength of inhibitory transmission from PV-INs to pyramidal neurons in PrL via MOR, and increases inhibitory synaptic transmission to PV-INs. **a** Schematic diagram indicating where *AAV-Flex-MOR-shRNA-EGFP* and *AAV-DIO-hChR2(H134R)-mCherry* were injected into PrL of *PV-Cre* or *SST-Cre* mice. **b** Representative confocal images showing the pyramidal neurons (PYR, Lucifer yellow) in PrL after whole-cell recordings upon optogenetic stimulation of PV- or SST-interneurons co-expressing *hChR2-mCherry* and *shRNA-EGFP*. Percentage of pyramidal neurons responsive to light-evoked activation of PV-INs (**c:** *n* = 30 cells/5 mice in saline/*Scramble* group, 33 cells/6 mice in morphine/*Scramble* group, 35 cells/6 mice in morphine/*MOR-shRNA* group; *χ*^2^ test) or SST-INs (**d**: *n* = 24 cells/4 mice in saline/*Scramble* group, 27 cells/4 mice in morphine/*Scramble* group, *n* = 23 cells/4 mice in morphine/*MOR-shRNA* group; *χ*^2^ test) 1 h after saline or 10 mg/kg morphine treatment. **e**–**h** Representative traces and responsive IPSC amplitudes onto pyramidal neurons from PV-INs (**e**, **f**: *n* = 27 cells/5 mice in saline/*Scramble* group, 29 cells/6 mice in morphine/*Scramble* group, 30 cells/6 mice in morphine/*MOR-shRNA* group; One-way ANOVA by the Bonferroni’s post-hoc test, *F*_(2,83)_ = 5.508, *P* = 0.0057) or SST-INs (**g**, **h**: *n* = 23 cells/4 mice in saline/*Scramble* group, 23 cells/4 mice in morphine/*Scramble* group, 22 cells/4 mice in morphine/*MOR-shRNA* group; One-way ANOVA by the Bonferroni’s post-hoc test, *F*_(2,65)_ = 1.505, *P* = 0.2297) 1 h after saline or morphine (10 mg/kg, i.p.) treatment in PrL slices expressing *Scramble* or *MOR-shRNA*. EYFP^+^ cells in PrL were recorded in acute slice from *SST-Cre::EYFP* or *PV-Cre::EYFP* mice 12 h after saline or morphine (10 mg/kg, i.p.) injection. Representative traces (**i**, **j**), cumulative probability distribution and average amplitude (**k**, **l**), and frequency (**m**, **n**) of mIPSCs recorded from SST-INs (*n* = 22–27 cells/4 mice in each group) and PV-INs (*n* = 36–40 cells/7–8 mice in each group; Mann–Whitney *U* test for the average and two-sample Kolmogorov–Smirnov test for cumulative probability). Representative AP traces and number of induced spikes in SST-INs (**o**, **p**) or PV-INs (**q**, **r**) (**o**, **p**: *n* = 35 cells/7 mice; current: *F*_(25,1700)_ = 191, *P* < 0.0001, treatment: *F*_(1,68)_ = 15.84, *P* = 0.0002, interaction: *F*_(25,1700)_ = 9.079, *P* < 0.0001; **q**, **r**: *n* = 29–30 cells/6 mice; current: *F*_(25,1425)_ = 192.9, *P* < 0.0001, treatment: *F*_(1,57)_ = 7.959, *P* = 0.0066, interaction: *F*_(25,1425)_ = 2.822, *P* *<* 0.0001; two-way RM ANOVA by the Bonferroni’s post-hoc test) in PrL after saline or morphine injection. Data are presented as mean ± S.E.M; *P < 0.05, **P < 0.01, ***P < 0.001, and ****P < 0.0001

To investigate morphine-induced disinhibition of pyramidal neurons mediated by different INs in PrL, PV-INs or SST-INs expressing *hChR2-H134R* were activated by serial laser pulses, and the responsive inhibitory postsynaptic currents (IPSCs) were measured in nearby pyramidal neurons (Fig. 1a, b). The responsive probability was comparable upon PV-INs or SST-INs activation (Fig. 1c, d). All light-evoked IPSCs could be abolished by 20 mM bicuculline (Fig. 1e, g). Morphine treatment (10 mg/kg, i.p.) significantly reduced the peak amplitude and the half-width of responsive IPSCs upon optogenetic activation of PV-INs, while did not change the 10–90% rise time or synaptic latency (Fig. 1e, f, and Supplementary Fig. 1a). Optogenetic activation of SST-INs in PrL evoked much weaker IPSCs in the nearby pyramidal neurons than activation of PV-INs, while the responsive probability and the peak amplitude of responsive IPSCs in pyramidal neurons were not changed by morphine exposure (Fig. 1g, h).

To investigate whether μ-opioid receptor (MOR) in the INs mediates the disinhibitory effect of morphine on PrL pyramidal neurons, we co-infected *AAV-DIO-(hChR2-H134R)-mCherry* with *AAV-Flex-MOR-shRNA-EGFP* or *AAV-Flex-Scramble-shRNA-EGFP* in the PrL of *PV-Cre* or *SST-Cre* mice (Fig. 1a). The downregulation of MOR in PV- and SST-INs was evaluated by immunostaining (Supplementary Fig. 2a–c). Laser evoked action potentials (APs) in PV-INs and SST-INs were not different between the *Scramble* or *MOR-shRNA* expressing cells (Supplementary Fig. 2d, e). Selective expression of *MOR-shRNA* in PV-INs abolished the inhibition by morphine on responsive IPSC amplitude in pyramidal neuron to laser stimulation of PV-INs (Fig. 1e, f). However, conditional knockdown of MOR in SST-INs had no effect on the response probability or the properties of the responsive IPSCs in pyramidal neurons to laser activation of SST-INs (Fig. 1d, g, h, and Supplementary Fig. 1b).

These results show that there is a broad connectivity between pyramidal neurons and PV- or SST-INs in the PrL, and PV-INs are able to evoke larger IPSCs in nearby pyramidal neurons than SST-INs. Morphine decreases the strength of synaptic inputs from PV-INs, but not SST-INs onto pyramidal neurons via a MOR-dependent pathway.

### Morphine increases neurite complexity of SST-INs and inhibitory transmission onto PV-INs in PrL

Addictive drugs are shown to regulate the density of dendritic spines and the electrophysiological activity of neurons in the mPFC [30]. To evaluate morphine-induced morphological changes in PrL INs, we used reporter mice (*SST-Cre::EYFP* and *PV-Cre::EYFP*) and injected a fluorescent dye into SST-INs and PV-INs for morphological tracing (Supplementary Fig. 3a). The results showed that 12 h after a single or five consecutive morphine injections, the neurite complexity (Sholl intersections) and total neurite length of SST-INs were significantly increased compared with the saline control group (Supplementary Fig. 3b, c). However, no significant difference in neurite complexity (Sholl intersections) and total neurite length in PV-INs were detected after morphine exposure (Supplementary Fig. 3d, e).

To examine synaptic transmission in these INs in PrL, we performed whole-cell patch-clamp recordings in PV-INs or SST-INs 12 h after saline or morphine exposure (Supplementary Fig. 4a). The amplitude of miniature excitatory postsynaptic currents (mEPSCs) recorded from SST-INs and PV-INs were both moderately decreased, while no difference in the frequency of mEPSCs was observed after morphine exposure (Supplementary Fig. 4b–e). Morphine exposure did not affect the amplitude or frequency of the miniature IPSCs (mIPSCs) in SST-INs (Fig. 1i, k, m), but increased both the frequency and the amplitude of mIPSCs in PV-INs (Fig. 1j, l, n), indicating that morphine enhances inhibitory inputs onto PV-INs in PrL. In addition, morphine increased the number of induced spikes in SST-INs (Fig. 1o, p), while decreased the number of induced spikes in PV-INs (Fig. 1q, r). The intrinsic electrophysiological characteristics of SST-INs and PV-INs did not change after morphine treatment (Supplementary Table 1).

These results suggest that morphine differentially regulates neurite complexity, inhibitory synaptic transmission, and membrane excitability of SST-INs and PV-INs in PrL.

### Morphine enhances the inhibitory synaptic transmission from SST-INs to fast-spiking (FS) PV-INs in PrL

**Fig. 2:**
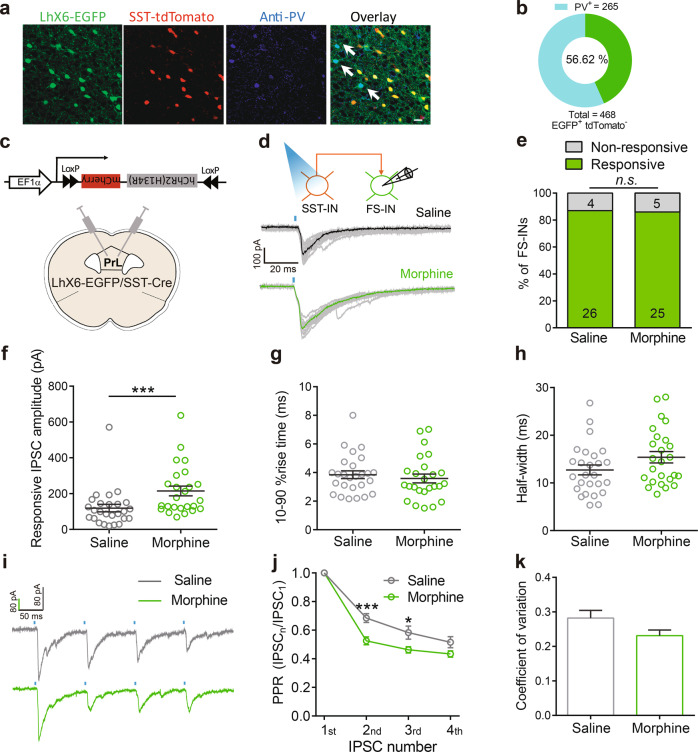
Morphine increases the strength of inhibitory transmission from SST-INs onto fast-spiking (FS) PV-INs in PrL. **a** Representative fluorescent images of a PrL coronal section from *LhX6-EGFP/SST-tdTomato* mice. Arrowheads indicate colocalization of PV antibody in EGFP^+^tdTomato^−^ cells. Scale bar, 20 μm. **b** Percentage of PV^+^ neurons in EGFP^+^tdTomato^−^ cells in PrL (265 PV^+^ cells in total 468 EGFP^+^tdTomato^−^ cells in eight slices from three mice). **c** Schematic diagram for *AAV-DIO-hChR2(H134R)-mCherry* injection into PrL of *LhX6-EGFP/SST-Cre* mice. **d** Representative traces of light-evoked response from SST-INs onto FS PV-INs in acute slice 12 h after saline or 10 mg/kg morphine injection. **e** Percentage of FS PV-INs responsive to optogenetic-activated SST-INs (*n* = 30 cells/five  mice in each group; *χ*^2^ test). Responsive IPSC amplitudes (**f**), the 10–90% rise time (**g**) and the half-width (**h**) of responsive IPSCs in FS PV-INs after saline or morphine exposure (*n* = 25–26 cells/five mice in each group; Mann–Whitney *U* test). **i** Representative traces of responsive amplitudes of IPSC responses in SST-INs to FS PV-INs connections. Light interval: 100 ms. Paired-pulse ratio (PPR) (**j**) and the coefficient of variation (**k**) of FS PV-INs in the PrL of mice injected with saline or morphine (*n* = 20 cells/five mice in each group; **j** two-way RM ANOVA by the Bonferroni’s post-hoc test. Number: *F*_(3,114)_ = 266.2, *P* < 0.0001, treatment: *F*_(1,38)_ = 8.944, *P* = 0.0049, interaction: *F*_(3,114)_ = 5.394, *P* = 0.0017; **k** Unpaired Student’s *t* test). Data are presented as mean ± SEM; n.s. not significant; *P < 0.05 and ***P < 0.001

Accumulating evidence indicates that the disinhibitory microcircuits in the cortex involve interactions among different subtypes of INs [31–34]. We thus investigated whether morphine regulates the strength of synaptic inputs from SST-INs onto PV-INs. We took advantage of *LhX6-EGFP* transgenic mice, in which the majority of INs derived from the medial ganglionic eminence are labeled with EGFP [35, 36], and bred this transgenic line with *SST::tdTomato* line (*LhX6-EGFP/SST-tdTomato* alleles) to distinguish SST-INs (tdTomato^+^) from other types of INs (EGFP^+^/tdTomato^−^) (Fig. 2a).

Immunostaining result showed that 56.62% of EGFP^+^/tdTomato^−^ INs is PV positive (Fig. 2b). Since FS is the most prominent electrical property of PV-INs, we recorded EGFP^+^/tdTomato^−^ INs with FS character. To examine synaptic transmission from SST-INs onto FS PV-INs, *AAV-DIO-(hChR2-H134R)-mCherry* was infected in the PrL of *LhX6-EGFP/SST-Cre* mice, and EGFP^+^/tdTomato^−^ FS-INs nearby tdTomato^+^ SST-INs were recorded upon optogenetic activation of SST-INs (Fig. 2c, d). The connection probability did not change after morphine exposure (Fig. 2e). The responsive amplitude, but not the 10–90% rise time or half-width of light-evoked IPSCs in FS-INs, was increased 12 h after morphine exposure (Fig. 2f–h), suggesting that morphine exerts long-lasting effect on the inhibitory synaptic transmission from SST-INs to FS-INs.

We next assessed the presynaptic release probability by analysis of paired-pulse ratio (PPR) and the coefficient of variation in FS PV-INs upon optogenetic stimulation of SST-INs. Morphine significantly reduced PPR ratio at the second and third stimulations (Fig. 2i, j). The coefficient of variation of responsive IPSCs did not significantly change (Fig. 2k). Combined with result of the increased mIPSCs frequency in PV-INs after morphine exposure, these data suggest that morphine increases the presynaptic release probability of SST-INs to PV-INs, and indicate that morphine-enhanced inhibitory GABAergic transmission from SST-INs onto PV-INs involves presynaptic mechanisms.

### Cell type-specific RNA-seq reveals that morphine upregulates Rac1 pathway specifically in SST-INs, but not PV-INs of PrL

**Fig. 3:**
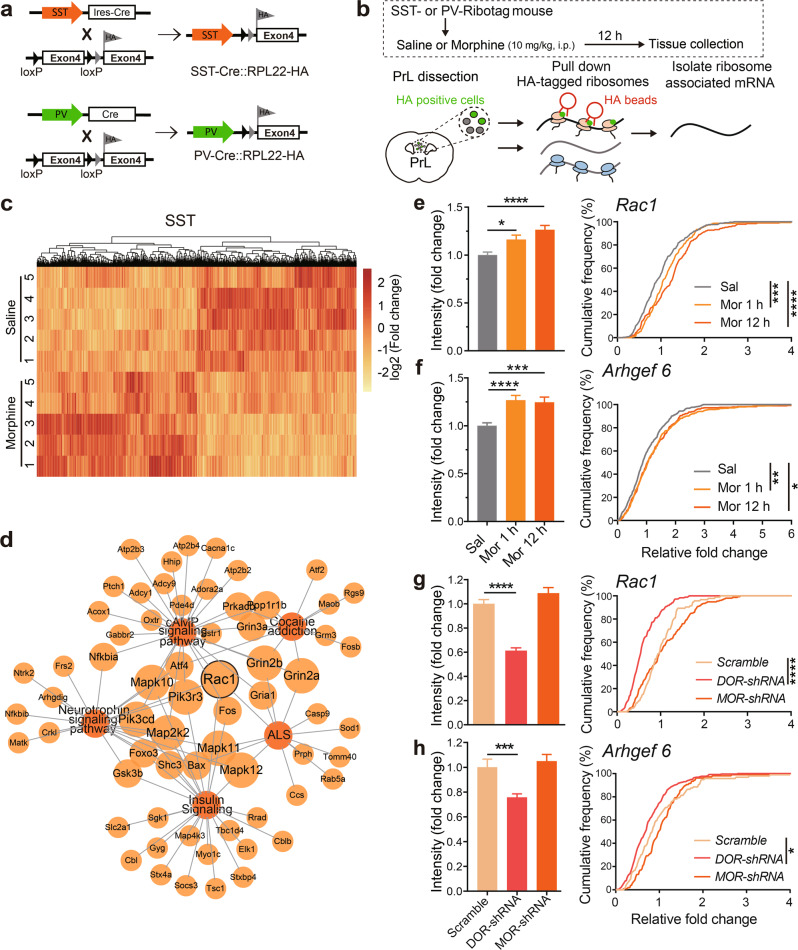
Morphine upregulates the expression of *Rac1* and *Arhgef6* in SST-INs via a DOR-dependent mechanism. Cell type-specific RNA-seq reveals morphine upregulates Rac1 pathway in SST-INs. **a** Breeding scheme of *SST-Cre::RPL22-HA* mice and *PV-Cre::RPL22-HA* mice. **b** Schematic procedure showing ribotag immunoprecipitation (IP) and RNA-seq of the ribosome-associated transcripts in PV-INs or SST-INs in PrL after saline or 10 mg/kg morphine injection. **c** The heat map of hierarchical clustering of normalized level of ribotag-isolated transcripts in SST-INs (five mice/group). Each row corresponds to a single gene. **d** Representation of the morphine-regulated signaling network enrichment analysis including all modules and contributing genes in SST-INs. **e**–**f** Single-molecule fluorescence ISH for *Rac1* or *Arhgef*6 transcript in SST-INs 1 h or 12 h after saline or morphine (10 mg/kg, i.p.) injection. Quantification of the fluorescent intensity of *Rac1* or *Arhgef6* transcripts in SST-INs. Three mice/group: *Rac1*: Sal, 247 cells, Mor 1 h, 150 cells, Mor 12 h, 192 cells; *Arhgef6:* Sal, 448 cells, Mor 1 h, 355 cells, Mor 12 h, 294 cells. **g**, **h** smFISH for *Rac1* or *Arhgef*6 in SST-INs expressing *DOR-shRNA, MOR-shRNA*, or *Scramble-shRNA* 12 h after morphine (10 mg/kg, i.p.) injection. Quantification of the fluorescent intensity of *Rac1* or *Arhgef6* transcripts in SST-INs expressing *shRNA*. 4 mice/group; *Rac1*: *Scramble*, 172 cells, *DOR-shRNA*, 185 cells, 154 cells; *Arhgef6*: *Scramble*, 125 cells, *DOR-shRNA*, 258 cells, *MOR-shRNA*, 106 cells. One-way ANOVA by the Bonferroni’s post-hoc test for intensity (**e:** *F*_(2,586)_ = 12.12, *P* < 0.0001; **f**: *F*_(2,1094)_ = 12.45, *P* < 0.0001; **g:** *F*_(2,508)_ = 52.05, *P* < 0.0001; **h**: *F*_(2,486)_ = 13.57, *P* < 0.0001). Two-sample Kolmogorov–Smirnov test for cumulative frequency. Data are presented as mean ± SEM; *P < 0.05, **P < 0.01, ***P < 0.001, and ****P < 0.0001

Given the observed difference of morphine-induced neuronal plasticity between SST-INs and PV-INs, the neuronal-specific molecular mechanisms between these two subtypes of neurons were assessed. *SST-Cre* and *PV-Cre* mice were crossed to *RPL22-HA* reporter mice to produce mice that express HA-tagged ribosomal protein (ribotag) specifically in SST- or PV-INs (Fig. 3a). Transcripts associated with ribosomes (in the process of de novo protein synthesis) were isolated from PrL 12 h after saline or morphine injection and sequenced (Fig. 3b). Analysis of the ribosome-associated transcripts in these two groups showed that *Pvalb* and *Sst* were respectively enriched in PV-INs and SST-INs (Supplementary Fig. 5a), indicating the successful enrichment of interneuron subtype-specific transcripts. Morphine injection induced more transcriptional alterations in SST-INs, compared with PV-INs (translational change in 1558 vs. 328 genes; Fig. 3c and Supplementary Fig. 5b).

To infer potential intracellular pathways altered by morphine, ClueGO was used for regulatory network construction of these two subtypes of INs. The analysis indicates that SST-INs exhibited significant differences in genes involved in the pathways of cAMP and insulin signaling, addiction, and neurotrophic factor signal transduction, etc., which have been associated with learning and memory, cognition, and neural plasticity. Consistent with the neurite complexity of SST-INs revealed by morphological study, Rac1, a member of the Rho family of GTPases and an important regulator of actin cytoskeleton and structural plasticity [37–39], was located on the hub section of the morphine-regulated signaling network in SST-INs (Fig. 3d). The changed ribosome-associated transcripts in PV-INs were enriched in the network including the insulin signaling, cell differential pathways, and protein–protein interaction, etc. (Supplementary Fig. 5c). qRT-PCR of ribosome-associated transcripts showed that the expression of the Rac1/Cdc42 guanine nucleotide exchange factor 6 (*Arhgef6*), and the immediate early genes such as *Arc* were upregulated in SST-INs (Supplementary Fig. 5d), but not in PV-INs (Supplementary Fig. 5e). These results indicate that morphine-induced morphological alterations in SST-INs couples with the changes of Rac1-related signaling pathways.

### Morphine upregulates the expression of *Rac1* and *Arhgef6* in SST-INs via DOR

Since the plasticity-related gene *Rac1* in SST-INs was identified as a hub gene in morphine-regulated signaling pathways, and the expression of *Arhgef6* was markedly upregulated in SST-INs after morphine exposure (Supplementary Fig. 5d), we performed single-molecule RNA in situ hybridization (ISH) to analyze the level of *Rac1* and *Arhgef6* transcripts in SST-INs. The results of RNAscope ISH showed that the intensity of *Rac1* and *Arhgef6* transcripts in SST-INs was upregulated 1 h after morphine injection and maintained at high level 12 h after the injection (Supplementary Fig. 6 a, b and Fig. 3e, f).

Morphine binds preferentially to MOR, while prolonged stimulation of neurons with morphine, both in vitro and in vivo, markedly increases recruitment of intracellular δ-opioid receptor (DOR) to the cell surface [40]. To explore the potential role of MOR and DOR in morphine-induced upregulation of *Rac1* and *Arhgef6* mRNAs in SST-INs, we infected *AAV-Flex-DOR-shRNA-EGFP* or *AAV-Flex-MOR-shRNA-EGFP* into PrL of *SST-Cre* mice. Cre-dependent *DOR* downregulation in SST-INs and PV-INs was verified by RNAscope ISH (Supplementary Fig. 7). We found that 12 h after morphine exposure, the fluorescent intensity of *Rac1* and *Arhgef6* transcripts was decreased in SST-INs expressing *DOR-shRNA*, whereas not changed in SST-INs expressing *MOR-shRNA* (Supplementary Fig. 6c, d and Fig. 3g, h). These results suggest that morphine enhances the expression of *Rac1* and *Arhgef6* in SST-INs via DOR, but not MOR.

### DOR and Rac1 in SST-INs mediate the enhancement of inhibitory transmission from SST-INs onto PV-INs by morphine and the disinhibition of pyramidal neurons in PrL

**Fig. 4:**
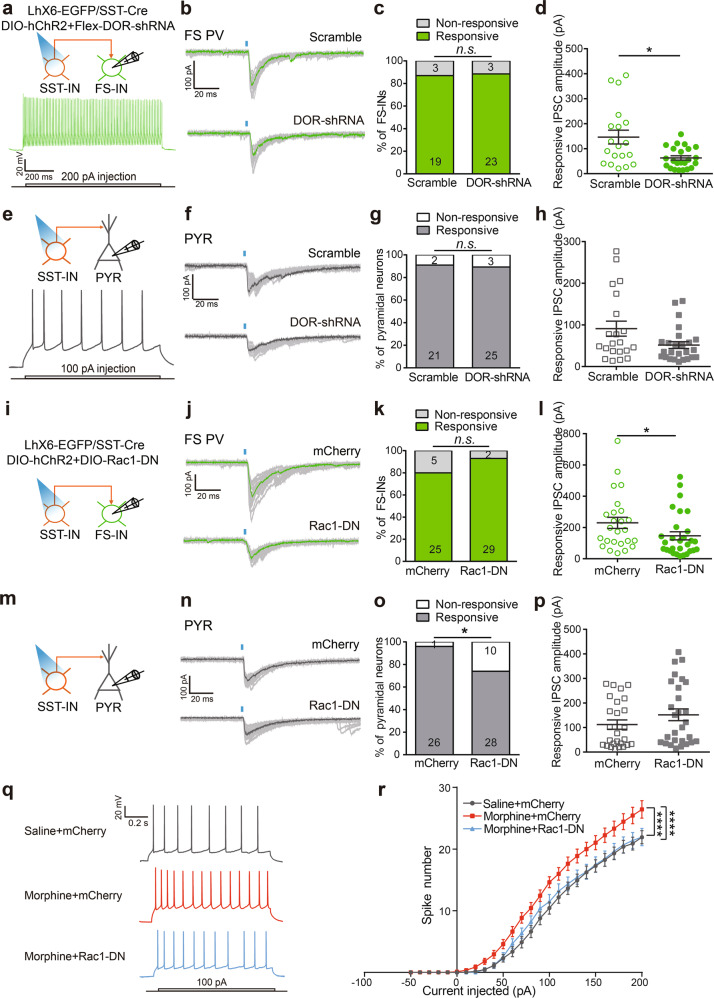
DOR and Rac1 in SST-INs are required for the enhancement of inhibitory transmission to FS PV-INs and the disinhibition of pyramidal neurons in PrL after morphine exposure. **a**–**h** *AAV-Flex-DOR-shRNA-EGFP* or *AAV-Flex-Scramble-shRNA* was co-injected with *AAV-DIO-hChR2(H134R)-mCherry* into the PrL of *LhX6-EGFP/SST-Cre* mice. **a** Schematic of the recording strategy and the spiking responses to intracellular current injection in FS PV-INs in acute slice 12 h after morphine exposure (10 mg/kg, i.p.). **b** Representative traces of the light-evoked response from SST-INs onto a FS PV-IN. **c** Quantification of the responsive probability from SST-INs onto a FS PV-IN in slice (*n* = 22–26 cells/5 mice in each group; *χ*^2^ test). **d** Quantitation of responsive IPSC amplitudes from SST-INs onto FS PV-INs (*n* = 19–23 cells from 5 mice/group; Mann–Whitney *U* test). **e** Schematic of the recording strategy and spiking responses to intracellular current injection in pyramidal neurons (PYR) in the same slice. **f** Representative traces of light-evoked response from SST-INs onto a pyramidal neuron. **g** Quantification of the responsive probability from SST-INs onto a pyramidal neuron in slice (*n* = 23–28 cells/5 mice in each group; *χ*^2^ test). **h** Quantitation of responsive IPSC amplitudes from SST-INs onto pyramidal neurons (*n* = 21–25 cells/5 mice in each group; Mann–Whitney *U* test). **i–p** *AAV-DIO-Rac1-DN-mCherry* or *AAV-DIO-mCherry* was co-injected with *AAV-DIO-hChR2(H134R)-mCherry* into the PrL of *LhX6-EGFP/SST-Cre* mice. **i** Schematic of the recording strategy in FS PV-INs in acute slice. **j** Representative traces of light-evoked response from SST-INs onto a FS PV-IN. **k** Quantification of the responsive probability from SST-INs onto a FS PV-IN in acute slice (*n* = 30–31 cells/7–8 mice in each group; *χ*^2^ test). **l** The quantitation of responsive IPSC amplitudes from SST-INs onto FS PV-INs (*n* = 25–29 cells/7 mice in each group; Mann–Whitney *U* test). **m** Schematic of the recording strategy in pyramidal neurons in the same slice. **n** Representative traces of light-evoked response from SST-INs onto a pyramidal neuron. **o** Quantification of the responsive probability from SST-INs onto a pyramidal neuron in slice (*n* = 27–38 cells/7–8 mice in each group; χ^2^ test). **p** Quantitation of responsive IPSC amplitudes from SST-INs onto pyramidal neurons (*n* = 26–28 cells/7–8 mice in each group; Mann–Whitney *U* test). **q**, **r** Representative recording of AP traces (**q**) and induced spikes (**r**) in pyramidal neurons nearby the SST-INs expressing mCherry or Rac1-DN after saline or morphine exposure (*n* = 26 cells/6 mice in Saline mCherry group, n = 32 cells/7 mice in Morphine mCherry group, 33 cells/7 mice in Morphine Rac1-DN group; two-way RM ANOVA by the Bonferroni’s post-hoc test. Saline mCherry vs. Morphine mCherry, current: *F*_(25,1400)_ = 503.1, *P* < 0.0001, treatment: *F*_(1,56)_ = 8.02, *P* = 0.0064, interaction: *F*_(25,1400)_ = 4.988, *P* *<* 0.0001; Morphine mCherry vs. Morphine Rac1-DN, current: *F*_(25,1575)_ = 452.3, *P* *<* 0.0001; virus: *F*_(1,63)_ = 5.106, *P* = 0.0273, interaction: *F*_(25,1575)_ = 3.244, *P* *<* 0.0001). Data are presented as mean ± SEM; n.s. not significant; *P < 0.05 and ****P < 0.0001

We further assessed whether morphine-enhanced inhibitory inputs onto PV-INs were mediated by DOR and Rac1 in SST-INs. We infected Cre-dependent *DOR-shRNA* and *hChR2-mCherry* viruses into the PrL of *LhX6-EGFP/SST-Cre* mice, and performed whole-cell recordings in FS PV-INs or pyramidal neurons nearby the opto-activated SST-INs 12 h after morphine exposure (Fig. 4a, e). Knockdown of DOR did not affect the light-evoked responsive probability in FS PV-INs (Fig. 4c), but decreased the responsive amplitude in FS PV-INs (Fig. 4b, d). In addition, knockdown of DOR did not affect the light-evoked responsive probability and the responsive amplitude of IPSCs in pyramidal neurons (Fig. 4f–h). These results suggest that knockdown of DOR in SST-INs decreased the strength of the inhibitory inputs from SST-INs to FS PV-INs.

We also constructed the *AAV-EF1α-DIO-Rac1-DN-mcherry* which expresses the dominant negative mutant of Rac1 (Rac1-DN) in a Cre-dependent manner. The neurite complexity and total neurite length of SST-INs or PV-INs were significantly decreased by the expression of Rac1-DN in PrL (Supplementary Fig. 8). Moreover, we infected Cre-dependent *Rac1-DN* and *hChR2-mCherry* viruses into the PrL of *LhX6-EGFP/SST-Cre* mice, and performed whole cell recordings in FS PV-INs or pyramidal neurons (Fig. 4i, m). Expressing Rac1-DN in SST-INs did not affect the light-evoked responsive probability in FS PV-INs (Fig. 4k), while attenuated the light-evoked responsive amplitude in PV-INs (Fig. 4j, l). However, expressing Rac1-DN in SST-INs decreased the light-evoked responsive probability (Fig. 4o), while did not affect the responsive amplitude in pyramidal neurons (Fig. 4n, p). In addition, pyramidal neurons nearby the SST-INs were significantly disinhibited by morphine, reflected by the increased current pulses, while the increased current pulses were abolished by expressing Rac1-DN in SST-INs (Fig. 4q, r). These data indicate that morphine increases the strength of the inhibitory transmission from SST-INs onto PV-INs via DOR and Rac1 signaling in SST-INs, and may thus attenuate the inhibitory effect of PV-INs onto pyramidal neurons in PrL.

### Distinct opioid receptor pathways in PrL SST-INs and PV-INs mediate morphine-conditioned place preference (CPP) and behavioral sensitization

**Fig. 5:**
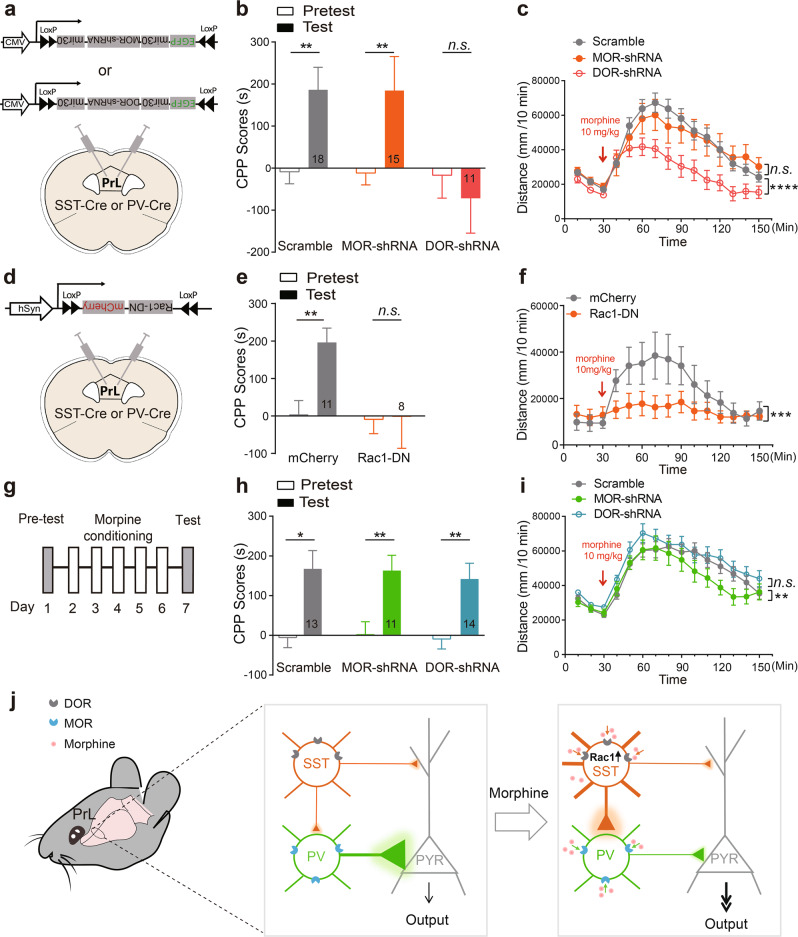
Distinct opioid receptors of SST-INs and PV-INs in the PrL coordinate morphine-induced CPP and behavioral sensitization. **a** Schematic of the PrL area where the *AAV-Flex-DOR-shRNA-EGFP*, *AAV-Flex-MOR-shRNA-EGFP* or *AAV-Flex-Scramble-shRNA-EGFP* was injected in *SST-Cre* or *PV-Cre* mice.The effect of downregulating DOR or MOR in SST-INs on morphine-induced CPP (**b:** *n* = 18 mice in *Scramble* group, 15 mice in *MOR-shRNA* group, 11 mice in *DOR-shRNA* group; Paired Student’s *t* test, *Scramble*, *P* *=* 0.0041, *MOR-shRNA*, *P* *=* 0.0095, *DOR-shRNA*, *P* *=* 0.6107) and hyper-locomotion (**c:** *n* = 24 mice in *Scramble* group, 12 mice in *MOR-shRNA* group, 13 mice in *DOR-shRNA* group; two-way RM ANOVA by the Bonferroni’s post-hoc test. *Scramble* vs. *DOR-shRNA*, time: *F*_(11,385)_ = 37.74, *P* < 0.0001, virus: *F*_(1,35)_ = 8.463, *P* = 0.0063, interaction, *F*_(11,385)_ = 5.586, *P* *<* 0.0001; *Scramble* vs. *MOR-shRNA*, time: *F*_(11,374)_ = 29.51, *P* < 0.0001, virus: *F*_(1,34)_ = 0.0693, *P* = 0.7940, interaction: *F*_(11,374)_ = 1.427, *P* *=* 0.1584). **d** Schematic of the PrL area where *AAV-DIO-Rac1-DN-mCherry* or *AAV-DIO-mCherry* was injected in *SST-Cre* or *PV-Cre* mice. The effect of downregulating Rac1 activity in SST-INs on morphine-induced CPP (**e:** *n* = 11 mice in mCherry group, 8 mice in Rac1-DN group; paired Student’s *t* test, mCherry, *P* *=* 0.0086, Rac1-DN, *P* *=* 0.9184) and hyper-locomotion (**f**: *n* = 8 mice in mCherry group, 12 mice in Rac1-DN group; two-way RM ANOVA by the Bonferroni’s post-hoc test. Time: *F*_(11,198)_ = 8.23, *P* < 0.0001, virus: *F*_(1,18)_ = 2.957, *P* = 0.1026, interaction, *F*_(11,198)_ = 4.973, *P* = 0.00029). **g** Experimental schedule for morphine-induced CPP. The effect of down-regulating DOR or MOR in PV-INs on morphine-induced CPP (**h**: *n* = 13 mice in *Scramble* group, 11 mice in *MOR-shRNA* group, 14 mice in *DOR-shRNA* group; paired Student’s *t*-test, *Scramble*, *P* *=* 0.0108, *MOR-shRNA*, *P* *=* 0.0049, *DOR-shRNA*, *P* *=* 0.0073) and hyper-locomotion (**i**: *n* = 20 mice in *Scramble* group, *n* = 10 mice in *MOR-shRNA* group, 12 mice in *DOR-shRNA* group; two-way RM ANOVA by the Bonferroni’s post-hoc test. *Scramble* vs. *MOR-shRNA*, time: *F*_(11,308)_ = 27.42, *P* < 0.0001, virus: *F*_(1,28)_ = 0.6785, *P* = 0.4171, interaction: *F*_(11,308)_ = 2.641, *P* *=* 0.0031; *Scramble* vs. *DOR-shRNA*, time: *F*_(11,330)_ = 29.33, *P* < 0.0001, virus: *F*_(1,30)_ = 0.8693, *P* = 0.3586, interaction: *F*_(11,330)_ = 1.119, *P* *=* 0.3449). Data are presented as mean ± SEM; n.s. not significant; *P < 0.05, **P < 0.01, ***P < 0.001, and ****P < 0.0001. **j** A model depicting the disinhibitory architecture in PrL and coordination by morphine. SST-INs (orange) innervate distal dendrites, while PV-INs (green) mainly target soma of pyramidal neurons (PYR), to exert distinct inhibitory effect on PYR in physiological state. Morphine attenuates the inhibitory input from PV-INs onto PYR via MOR (blue), while upregulates the Rac1 in SST-INs via DOR (gray) to enhance its inhibitory effect onto PV-INs. This architecture specifically coordinated by morphine via different opioid receptors disinhibits pyramidal neurons in PrL, and thus enhances reward

Prefrontal cortex has been implicated in the reward processing and the development of addictive-drug-induced behavioral sensitization [3]. To investigate the effect of MOR and DOR signaling pathways in specific INs on the rewarding properties and locomotor-activating effects of morphine, *SST-Cre* and *PV-Cre* mice were infected with *AAV-Flex-MOR-shRNA-EGFP*, *AAV-Flex-DOR-shRNA-EGFP* or *AAV-Flex-Scramble-shRNA-EGFP* in PrL (Fig. 5a). Results showed that knockdown of DOR in SST-INs significantly inhibited morphine-induced CPP and hyper-locomotion, while knockdown of MOR in SST-INs had no such effects (Fig. 5b, c). We then injected *AAV-EF1α-DIO-Rac1-DN-mCherry* or *AAV-EF1α-DIO-mCherry* into the PrL of *SST-Cre* and *PV-Cre* mice (Fig. 5d). Expressing Rac1-DN in SST-INs abolished morphine-induced CPP and hyper-locomotion (Fig. 5e, f), while expressing Rac1-DN in PV-INs had no effect (Supplementary Fig. 9). These results indicate that DOR-Rac1 pathway in the SST-INs, but not in the PV-INs, is involved in the reward properties and locomotor-activating effects of morphine.

Knockdown of MOR in PV-INs had no effect on morphine-induced CPP, but decreased locomotor activity 90 min after the initial morphine injection (Fig. 5h, i) and behavioral sensitization after repeated morphine exposures (Supplementary Fig. 10), suggesting that MOR of PV-INs in PrL is involved in both the initiation and expression of behavioral sensitization to morphine. Knockdown of DOR in PV-INs had no effect on morphine-induced CPP and hyper-locomotion (Fig. 5h, i).

Taken together, these data reveal that DOR-Rac1 pathway in SST-INs is required for morphine-induced CPP and hyper-locomotion, while MOR pathway in PV-INs is involved in behavioral sensitization, and indicate that morphine, via distinct opioid receptors, coordinates an architecture consisting of SST-INs and PV-INs to disinhibit pyramidal neuron in PrL and enhance reward.

## Discussion

GABAergic INs, as a minority of the cortical neuronal population in the forebrain, are crucial in fine-tuning cortical microcircuits. The interactions of GABAergic INs with excitatory glutamatergic neurons maintain balanced electrical activity and normal cortical functions [41]. Morphine activates presynaptic GABAergic neurons in VTA and disinhibits dopaminergic neurons, increasing dopamine release and inducing reward [42]. However, the molecular targets and the inhibitory architecture recruited by morphine to promote reward and behavioral sensitization are unclear.

PV-INs includes FS basket and chandelier cells. FS-INs are the largest population of INs in the neocortex. They regulate action potential firing and form complex structural contacts between themselves to promote synchronization of electrical activity [14, 43, 44]. In contrast to PV-INs, SST-INs are dendritic targeting and they mediate double-synapse inhibition on nearby pyramidal neurons [45]. We examined the amplitude of responsive IPSC from PV-INs and SST-INs onto pyramidal neurons by optogenetic stimulation, and observed that as compared with SST-INs, PV-INs showed stronger predominant inhibitory inputs onto pyramidal neurons (Fig. 1). SST-INs showed an increased intrinsic membrane excitability, while PV-INs showed a decreased intrinsic membrane excitability upon morphine exposure. Consistently, SST-INs exhibited more translational and morphological changes upon morphine exposure (Fig. 3 and Supplementary Fig. 3), indicating that morphine-induced alterations are interneuron subtype-specific and likely via differential modulatory mechanisms.

SST-INs not only innervate pyramidal neurons but also strongly innervate other types of INs in the cortex [33, 34, 46]. The increased frequency of mIPSC in PV-INs indicates an increased inhibitory input. Utilizing *LhX6-EGFP/SST-tdTomato* mice, we identified PV-INs by EGFP^+^/tdTomato^−^ labeling and FS features to study the synaptic transmission from SST-INs to FS PV-INs. Our data suggest that morphine exposure increases the inhibitory transmission from SST-INs to FS PV-INs in PrL and this is dependent on a presynaptic mechanism. We postulate that after morphine stimulation, SST-INs extend their neurites to form extensive reciprocal connections and stronger inhibition to adjacent FS PV-INs, and this is accompanied with reduced FS PV-INs firing, leading to disinhibition of nearby pyramidal neurons and behavioral changes.

Morphine exhibits affinity to MOR, DOR, and KOR subtypes, but has higher affinity to MOR and thus preferentially binds to MOR [47]. The results from MOR- and DOR-knockout mice indicate that MOR is essential for both of analgesia and tolerance of morphine [48], while DOR is required for the development of sensitization and tolerance to the locomotor-activating effects of morphine [49]. Previous histological data showed that MORs are expressed prominently in PV-INs, whereas DORs are expressed prominently in SST, neuropeptide Y, and corticotrophin releasing factor (CRF) INs, as well as in pyramidal neurons in hippocampus [50]. It is interesting to know how morphine remodels the circuits to promote reward by activating signaling pathways mediated by different opioid receptors in different INs.

In this study, we focus on MOR and DOR signaling and the inhibitory transmission from PV- and SST-INs onto pyramidal neurons in PrL. Our results reveal that morphine exerts its disinhibition function on pyramidal neurons via neuronal subtype- and opioid receptor-specific signaling pathways. The activation of MOR-signaling pathway in PV-INs by morphine attenuates the inhibitory inputs to pyramidal neurons directly, while activation of DOR signaling pathway in SST-INs by morphine enhances the inhibitory inputs to PV-INs and thus further disinhibits pyramidal neurons nearby (Fig. 5j). Our results showed that morphine specifically increases the neurite complexity and upregulates *Rac1* and *Arhgef6* in SST-INs, while knockdown of DOR in SST-INs decreased the mRNA level of *Rac1* and *Arhgef6*. Knockdown of Rac1 in SST-INs abolished morphine-induced strengthening of the inhibitory inputs to FS PV-INs and the increase of activity of the pyramidal neuron. These data indicate that the Rac1 is downstream of opioid receptor in SST-INs, and it mediates the inhibition of SST-INs on nearby PV-INs.

MOR in PV-INs and DOR in SST-INs in PrL mediate morphine-induced CPP and behavioral sensitization, respectively, indicating that morphine-induced reward processing and behavioral sensitization require the activation of neuronal-specific opioid receptors in PrL. Our results suggest that the acute effect of morphine to disinhibit pyramidal neurons is via attenuating the inhibitory synaptic transmission from PV-INs to pyramidal neurons, while the long-lasting disinhibition effect of morphine (12 h after morphine exposure) on pyramidal neurons is through enhancing the strength of the inhibitory transmission from SST-INs onto PV-INs. Since morphine has higher affinity for MOR than DOR, we hypothesize that morphine initially activates the MORs in the PV-INs, directly inhibits PV-INs producing acute disinhibition of pyramidal neurons and behavioral sensitization, and excessive morphine activates DORs in SST-INs, thus inducing prolonged inhibition on PV-INs via upregulation of Rac1, blocking the inhibitory inputs from PV-INs to the PrL pyramidal neurons and mediates CPP.

Research on the structural and functional connectivity between inhibitory INs and pyramidal neurons is important for understanding on how inhibitory architecture in PrL gates neuronal network excitability. The circuitry for behavioral sensitization includes glutamatergic projections from the mPFC to the NAc [3, 51], and the prefrontal glutamate release into NAc mediates drug-seeking behaviors [52]. The output circuits of PrL guide conditioned reward seeking through divergent PFC → NAc and PFC → PVT encoding [53]. mPFC → NAc population dynamics predict individual reward seeking or suppression decision [54]. These results indicate that glutamatergic projection in PrL, which is modulated precisely by local INs, is required for behavioral sensitization and reward. Our data indicate that morphine remodels SST- and PV-interneuron plasticity, which in turn induces behavioral changes via distinct molecular pathways in the two types of INs. The MOR and DOR signaling pathways in the INs play important regulatory roles in behavioral sensitization and reward processing.

## Materials and methods

### Animals

*SST-Cre* mice (013044), *PV-Cre* mice (012358), *Rosa 26* reporter mice (006148), *Ai14* reporter mice (007914), and Ribotag mice (011029) were purchased from The Jackson Laboratory (CA, USA). *LhX6-EGFP* mice (000246-MU) were purchased from Mutant Mouse Resource & Research Centers (MMRRC). These mice were bred to C57BL/6 J for more than 6 generations. *SST-Cre::EYFP* or *PV-Cre::EYFP* alleles were generated by crossing *SST-Cre* or *PV-Cre* mice with *Rosa 26* reporter mice; *SST-Cre::RPL22-HA* or *PV-Cre:: RPL22-HA* alleles were generated by crossing *SST-Cre* or *PV-Cre* mice with Ribotag mice*; SST-Cre::tdTomato* alleles were generated by crossing *SST-Cre* mice with *Ai14* mice; *LhX6-EGFP*/*SST-Cre::tdTomato* mice were generated by crossing *LhX6-EGFP* mice with *SST-Cre::tdTomato* mice; *LhX6-EGFP/SST-Cre* were generated by crossing *LhX6-EGFP* mice with *SST-Cre* mice. 6–10-week-old male offsprings were used in the experiments, and randomly assigned to groups. Mice used for the experiments were housed in groups on a 12 h light/dark cycle (light on from 8 a.m. to 8 p.m.) with access to food and water ad libitum. All experiment procedures were strictly in accordance with the National Institutes of Health Guide for the Care and Use of Laboratory Animals, and were approved by Animal Care and Use Committee of the animal facility at Fudan University.

### Viral constructs

Fragment encoding Rac1 dominant negative mutant (Rac1-DN, T17N) [55] was subcloned into *pAAV-EF1α-DIO-mCherry* using AscI/NheI restriction sites to yield *pAAV-EF1α-DIO-Rac1-DN-mcherry*. For Cre-dependent expression of *shRNAs* in cells and transgenic mice, the *shRNAs* coding sequence targeting mouse MOR (5′-CGGCTAATACAGTGGATCGAA-3′) or DOR (5′-GTGCTATGGCCTCATGCTACT-3′) were cloned into the *pAAV-CMV-Flex-MIR30shRNA-EGFP* vector (Obio Technology, Shanghai, China) using EcoRI/XhoI restriction sites. *AAV*_*9*_*-EF1α-DIO-Rac1-DN-mcherry, AAV*_*9*_*-Flex- MOR-shRNA-EGFP, AAV*_*9*_*-Flex-DOR-shRNA-EGFP* or *AAV*_*9*_*-Flex-Scramble-shRNA-EGFP* viruses were packaged by Obio Technology (Shanghai, China). *AAV*_*9*_*-EF1α-DIO-mCherry* and *AAV*_*9*_*-EF1α-DIO-hChR2(H134R)-mCherry* were purchased from Taitool Bioscience (Shanghai, China).

### Stereotaxic surgery

Mice were anesthetized with 2% isoflurane and placed in a stereotactic instrument (Stoelting, Kiel, WI, USA). Microinjections were performed using 33-gauge needle connected to a 10 μl Hamilton syringe. The intended stereotaxic coordinates for PrL were: AP + 2.0 mm; ML ± 0.3 mm (with an angle of 14° from the middle to the lateral); DV —2.0 mm. Each site was injected with 0.5 μl of purified and concentrated AAV (10^12^ IU/ml) with a slow injection rate (0.1 μl/min). All mice were given at least 3 weeks to recover before behavioral experiments or electrophysiological recordings, and the efficiency of viral infection and the shRNA knockdown was verified by immunostaining. The histology slides were examined blindly to check the expression of EGFP or mCherry in PrL. Only the mice with virus infection in correct place were chosen for further analysis.

### Brain slice preparation and electrophysiological recording

Coronal sections (300 μm) containing PrL were prepared as previously described [56]. Briefly, the mice were anesthetized by isoflurane and then transcardially perfused with cold artificial cerebrospinal fluid [ACSF; 92 mM N-methyl-D-glucamine, 2.5 mM KCl, 1.25 mM NaH_2_PO_4_, 30 mM NaHCO_3_, 20 mM HEPES, 25 mM d-glucose, 2 mM thiourea, 5 mM Na-ascorbate, 3 mM Na-pyruvate, 0.5 mM CaCl_2_, and 10 mM MgCl_2_]. Brains were quickly removed, sliced with the vibratome (Thermo Scientific, MA, USA) and incubated in protective ACSF saturated with 95% O_2_, 5% CO_2_, and the slices were used within 6 h after preparation. Individual neurons were identified under a BX51WI microscope (Olympus, Tokyo, Japan) equipped with Rolera Bolt CCD camera (QImaging, Surrey, BC, Canada). Whole-cell voltage clamp recordings were performed in oxygenated ACSF (124 mM NaCl, 2.5 mM KCl, 1.2 mM NaH_2_PO_4_, 24 mM NaHCO_3_, 5 mM HEPES, 12.5 mM glucose, 2.4 mM CaCl_2_, and 1.2 mM MgCl_2_) at 31~32 °C with an EPC-10 amplifier and Patchmaster software (HEKA Elektronik, Lambrecht/Pfalz, Germany). The pipette resistance was in the range of 8–10 MΩ. Current clamp recordings were filtered at 2.9 kHz and sampled at 5 kHz.

For light-evoked postsynaptic currents whole-cell recordings, ChR2 was excited with a 473 nm LED blue light source (Xcite-110) delivered through the epifluorescence pathway and fed into a 60× water-immersion objective lens (Olympus BX51). Photo-stimulation (2–6 mW/mm^2^, 1–2 ms duration) was controlled by a TTL input (HEKA Instruments). Pyramidal neurons or FS INs were clamped at −70 mV. For recordings, the pipettes were filled with intracellular solution (60 mM K-gluconate, 66 mM KCl, 2 mM MgCl_2_, 10 mM HEPES, 0.2 mM EGTA, 4.5 mM MgATP, 0.5 mM Na_3_GTP, 10 mM Na-phosphocreatine, 0.25% neurobiotin, pH 7.25, 300 mOsm). D-APV (100 mM) and CNQX (20 mM) were added to block excitatory currents. A train of light pulses (10 Hz) was delivered to the presynaptic cells, and the postsynaptic responses were recorded 20–30 repeated trails at 15 or 20 s interval. Data were analyzed off-line with Clampfit 10.3 (Molecular Devices, Union City, CA, USA) or Mini Analysis Program (Synaptosoft, Fort Lee, NJ, USA). The peak amplitude was calculated by subtracting the baseline. The synaptic latency was determined as the duration from current onset time to peak time. The rise time of evoked IPSCs was assessed from the 10 to 90% rising phase, and the half-width of evoked IPSCs was defined as the duration at the half amplitude. For the paired-pulse ratio calculation, the averaged peak amplitude of the first IPSC was defined as the basal level of synaptic strength. The variable coefficient was assessed from the amplitudes of each sweep.

We used modified intracellular solution (127.5 mM cesium methanesulfonate, 7.5 mM CsCl, 10 mM HEPES, 2.5 mM MgCl_2_, 4 mM Na_2_ATP, 0.4 mM Na_3_GTP, 10 mM sodium phosphocreatine, 0.6 mM EGTA, pH 7.25, 290 mOsm) to adjust the reversal potential of the γ-aminobutyric acid-A receptor (GABAaR) response. mEPSC events were recorded in the presence of 2 μM TTX and GABAaR blocker (bicuculline methiodide, 10 μM) (Tocris Bioscience, Bristol, UK), at a holding potential of −60 mV. mIPSC events were recorded in the presence of 2 μM TTX, NMDA receptor blocker (10 μM D-APV), and AMPA receptor blocker (CNQX, 20 μM), at a holding potential of +10 mV. The intracellular solution (130 mM K-gluconate, 6 mM KCl, 2 mM MgCl_2_, 10 mM HEPES, 2.5 mM ATP-Mg, 0.5 mM GTP-Na_2_, 10 mM creatine phosphate, 0.6 mM EGTA, pH 7.25, 290 mOsm) was used for recording action potential. After achieving whole cell configuration, a current-step protocol (from −200 to +200 pA, with 10 pA increment) was run and repeated. Recordings with *R*_s_ > 30 MΩ were excluded from statistical analysis. Data were filtered at 300 Hz and were analyzed by Mini Analysis Program (Synaptosoft).

### Morphological reconstruction and quantitation

For neuronal reconstruction and morphological analysis, SST and PV INs in PrL were randomly selected, patched, and filled with 2% lucifer yellow (L0259, Sigma-Aldrich, St Louis, WA, USA) for at least 10 min. After 10 min additional diffusion, slices were fixed in 4% PFA in 0.1 M phosphate-buffered saline (PBS) overnight. Sections were blocked in 10% serum and 0.1% Triton-X in PBS, and incubated with an anti-Lucifer Yellow antibody (Invitrogen, #A-575c, Carlsbad, CA, USA) overnight at 4 °C. Z-series images were taken at 2 μm interval using an Olympus FV1000 confocal laser scanning microscope with a 60× objective (Olympus). Full cell 3-dimensional reconstructions and analysis were made by Neurolucida (MicroBrightField, Williston, VT, USA).

### Immunohistochemistry

Mice were anesthetized with isoflurane and perfused with saline followed by 4% paraformaldehyde in 0.1 M PBS. The brains were removed, fixed in 4% paraformaldehyde overnight and subjected to dehydration in 30% sucrose at 4 °C for 72 h before slicing 30 μm per slice. Slices were incubated with diluted antibodies in blocking solution containing 0.2% Triton X-100 (Sigma-Aldrich) and 3% goat serum (Jackson ImmunoResearch, West Grove, PA, USA) at 4 °C overnight. The primary antibodies used were: anti-SST (Santa Cruz, #sc-47706, Dallas, TX, USA), anti-PV (Merck Millipore, #MAB1572, Darmstadt, Germany) and anti-MOR (Abcam, #ab10275, Cambridge, MA, USA). Slices were rinsed in 0.1 M PBS then incubated in Cy3 anti-mouse, Alexa 647 anti-rat or Cy3 anti-rabbit IgG antibodies (Jackson ImmunoResearch) for 1 h at room temperature, then mounted after rinsing with 0.1 M PBS. Images were acquired on a Nikon A1 microscope (Tokyo, Japan) using 20× air or 60× oil objective lens. The observer analyzing the expression of MOR in SST or PV INs was blinded to the group allocation.

### RNAscope ISH

The frozen brain tissue was sliced into 10 μm coronal sections and mounted onto Colorfrost Plus slides (ThermoFisher, Waltham, MA, USA). Slices were incubated with hydrogen peroxide 10 min RT, target-retrieval solution and Protease III using RNAscope^®^ 2.5 Universal Pretreatment Reagents (Advanced Cell Diagnostics, #322380, Newark, CA, USA). smFISH for all genes examined, *Rac1* (#517461), *Arhgef6* (#574371), *EGFP* (#400281-C3), *Sst* (#404631-C2), *Pvalb* (#421931-C2), and *Oprd1* (#427371-C3) were performed hybridization for 2 h. After hybridization, we used the RNAscope^®^ Multiplex Fluorescent Detection Kit v2 (#323110) to amplify signal and mounted. Images were acquired with a Nikon A1 microscope using 20× objective. IOD in SST^+^ or PV^+^ neurons was analyzed by Image-Pro Plus 6.0 (Media Cybernetics, Rockville, MD, US). The observer analyzing the expression of *DOR, MOR,* *Rac1*, and *Arhgef6* in SST, or PV INs was blinded to the group allocation.

### Ribo-tag purification

Purification of ribosome-associated mRNA was performed as described previously with slight modification [57]. Mice were decapitated, and the brains were removed immediately. The PrL were dissected in ice-cold PBS. The brain tissue was homogenized in 1 ml Supplemented Hybridization Buffer (25 mM Tris pH 7.0, 25 mM Tris pH 8.0, 12 mM MgCl_2_, 100 mM KCl, 1% Triton X-100) containing 1 mM DTT, 1 × protease inhibitors (Roche, Upper Bavaria, Germany), 200 U/ml RNase inhibitor (Promega, Madison, WI, USA), 100 μg/ml cycloheximide (Cayman, Ann Arbor, MI, USA), and 1 mg/ml heparin (Sigma-Aldrich). The supernatant was incubated with 10 μg anti-HA antibody (Sigma-Aldrich, #H6908) and 100 μl Dynabeads Protein G (Invitrogen) for 12 h. Purified mRNA was eluted from the Dynabeads using TRIzol LS (Invitrogen) according to the manufacturer’s instructions with the inclusion of a DNase digestion step. The Agilent RNA 6000 Pico Kit (Agilent, Santa Clara, CA, USA) and Agilent 2100 bioanalyzer were used to evaluate the quality of purified mRNA. Samples with RIN number > 7 were used.

### Next-generation sequencing

mRNA was enriched using NEB Next Poly(A) mRNA Magnetic Isolation Module (NEB, E7490S, Ipswich, MA, USA). Library was prepared with NEB Next Ultra RNA Library Prep Kit (E7530S) and sequenced on a HiSeq 4000 (Illumina) by Novogene Technology Co. Ltd (Beijing, China). Raw reads were quality checked and trimmed with FASTX-toolkit to remove adapter contamination and low-quality reads (quality score < 28). The clipped reads were aligned to mouse reference sequence (GRCm38/mm10) using HISAT2. Mapped reads for each transcript were counted using *HTseq* and differential expression analysis was performed with *DESeq2*. Genes with more than twofold expression changes, and were significantly different (*P* < 0.05) were selected for further analysis. ClueGo [58] was used for signaling pathway and network construction.

### Reverse transcription, and quantitative real-time PCR (qRT-PCR)

Reverse transcription was completed using the PrimeScript RT reagent Kit (RR037A, Takara Biotechnology, Dalian, China). The cDNA was subjected to qRT-PCR using SYBR Premix Ex Taq (RR420A, Takara) and Eppendorf Mastercycler PCR System (Eppendorf, Hamburg, Germany). The primers are listed in Supplementary Table 2.

### Locomotion test

An activity monitor system (43.2 cm length × 43.2 cm width × 30.5 cm height, Med-Associates, St. Albans, VT, USA) was used to detect morphine-induced locomotor activities and behavioral sensitization. Each mouse was placed in the center of the open field and allowed to explore freely for 30 min (baseline). After given an intraperitoneal injection of morphine (10 mg/kg) (Shenyang 1st Pharmaceutical Company, Shenyang, China), the mice were confined to the open field for 120 min. To evaluate morphine-induced behavioral sensitization, mice were placed in chamber for 10 min, then injected with morphine (10 mg/kg, i.p.) and placed in chamber for 1 h. The total distance traveled was recorded.

### Conditioned place preference

Morphine-induced CPP was performed using a two chamber (15 × 15 × 20 cm) apparatus with distinct tactile environments to maximize contextual differences. A manual guillotine door (15 × 20 cm) separated the two chambers. The observer was blinded to the group allocation. On the first day, mice were allowed to freely explore the entire apparatus for 15 min (pretest). The mice staying in one chamber for more than 10 min were excluded from the experiment. From the second to the sixth days, mice were daily given an intraperitoneal injection of morphine (10 mg/kg, i.p.) and confined to one of the chambers (drug-paired) for 30 min, and 6 h later, they received an i.p. injection of saline (equivalent volume to that of morphine) and confined to the other chamber for 30 min (conditioning). On the seventh day, mice were allowed to freely explore the entire apparatus for 15 min (test). The time spent in each chamber was recorded during the pretest and test sessions. CPP score was defined as the time (in seconds) spent in morphine-paired chamber minus the time spent in saline-paired chamber.

### Statistical analysis

Data were analyzed with SPSS 20 software (IBM, Armonk, NY, USA). Sample size estimation was conducted on alpha value of 0.05 and desired power of 0.80. Comparisons between groups were made by unpaired or paired two-tailed student’s *t* test, Mann–Whitney *U* test, *χ*^2^ test, one-way ANOVA, or two-way ANOVA. Two-sample Kolmogorov–Smirnov test was used for analyzing the cumulative distribution. Results of locomotion and neuronal excitability were analyzed by two-way repeat-measure (RM) ANOVA followed by the Bonferroni’s post-hoc test. Statistical significance was represented as *P *<* 0.05; **P *<* 0.01; ***P *<* 0.001, and ****P *<* 0.0001. All data are presented as mean ± SEM.

## Supplementary information

Supplementary Material

## Data Availability

All data needed to evaluate the conclusions in the paper are present in the paper and/or the supplementary materials. Additional data related to this paper may be requested from the authors. Raw and processed NGS data are deposited in the National Center for Biotechnology Information BioProject database under accession number (PRJNA508422).
