# Supplementary material for: Morphine coordinates SST and PV interneurons in the prelimbic cortex to disinhibit pyramidal neurons and enhance reward
Source: Mol Psychiatry. 2019 Aug 14;26(4):1178–93. doi: 10.1038/s41380-019-0480-7 (PMC7985023; doi:10.1038/s41380-019-0480-7)
Supplement: Supplementary file 1 — Supplementary Material [file 41380_2019_480_MOESM1_ESM.pdf]

Supplementary Material

Figure S1. Quantification of the basic waveform parameters of the responsive IPSC.

Figure S2. Knockdown efficiency of *MOR-shRNA,* and light-evoked AP firing in SST-INs or PV-INs expressing S*cramble* or *MOR-shRNA*.

Figure S3. Morphine increases neurite complexity in SST-INs.

Figure S4. Morphine decreases the amplitude of mEPSC in SST-INs and PV-INs.

Figure S5. Expression profiling of the ribosome-associated transcripts reveals the enrichment of cell type-specific markers, and qRT-PCR verification of the morphine-changed transcripts in SST-INs and PV-INs.

Figure S6. Representative confocal images of smFISH showing the distribution of *Rac1* or *Arhgef*6 transcripts in SST-INs.

Figure S7. Verification of the knockdown efficiency of *DOR-shRNA* in SST-INs and PV-INs.

Figure S8. The effect of Rac1-DN on the morphology of SST-INs and PV-INs.

Figure S9. The effect of expressing Rac1-DN in PV-INs on morphine-induced CPP and hyper-locomotion.

Figure S10. The effect of knockdown of MOR in PV-INs on morphine-induced behavioral sensitization.

Table S1. Intrinsic electrophysiological properties of SST-INs and PV-INs in PrL.

Table S2. DNA sequence of qRT-PCR primers.

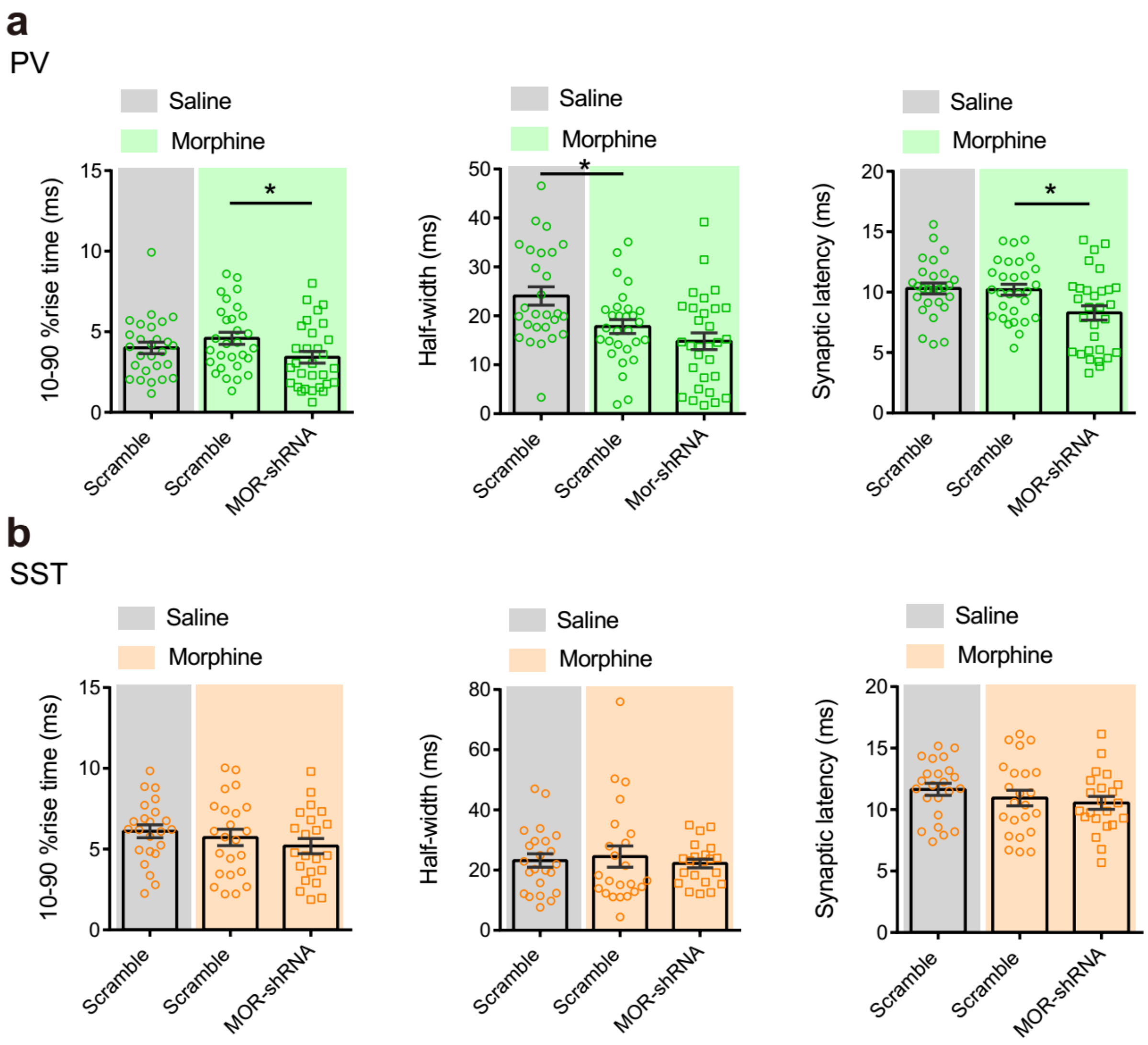

Fig. S1. Quantification of the basic waveform parameters of the responsive IPSC.

a Quantification of the 10-90 % rise time (left), half-width (middle), and synaptic latency (right) of responsive IPSCs in PV-INs (n = 27 cells/5 mice in saline/*Scramble* group, n = 29 cells/6 mice in morphine/*Scramble* group, n = 30 cells /6 mice in morphine/*MOR-shRNA* group; One-way ANOVA by Bonferroni's *post-hoc* test. Left: $F_{(2, 83)} = 2.648$,$P = 0.0767$; middle: $F_{(2, 83)} = 7.826$, $P = 0.0008$; right: $F_{(2, 83)} = 5.124$, $P = 0.0080$). **b** Quantification of the 10-90 % rise time (left), half-width (middle), and synaptic latency (right) of responsive IPSCs in SST-INs (n = 23 cells/4 mice in saline/*Scramble* group, n = 23 cells/4 mice in morphine/*Scramble* group, n = 22 cells/4 mice in morphine/*MOR-shRNA* group; One-way ANOVA by Bonferroni's *post-hoc* test

test. Left: $F_{(2, 65)} = 0.9778$, $P = 0.3816$; middle: $F_{(2, 65)} = 0.1967$, $P = 0.8220$; right: $F_{(2, 65)} = 1.007$, $P = 0.3709$). Data are presented as mean ± S.E.M. *$P < 0.05$.

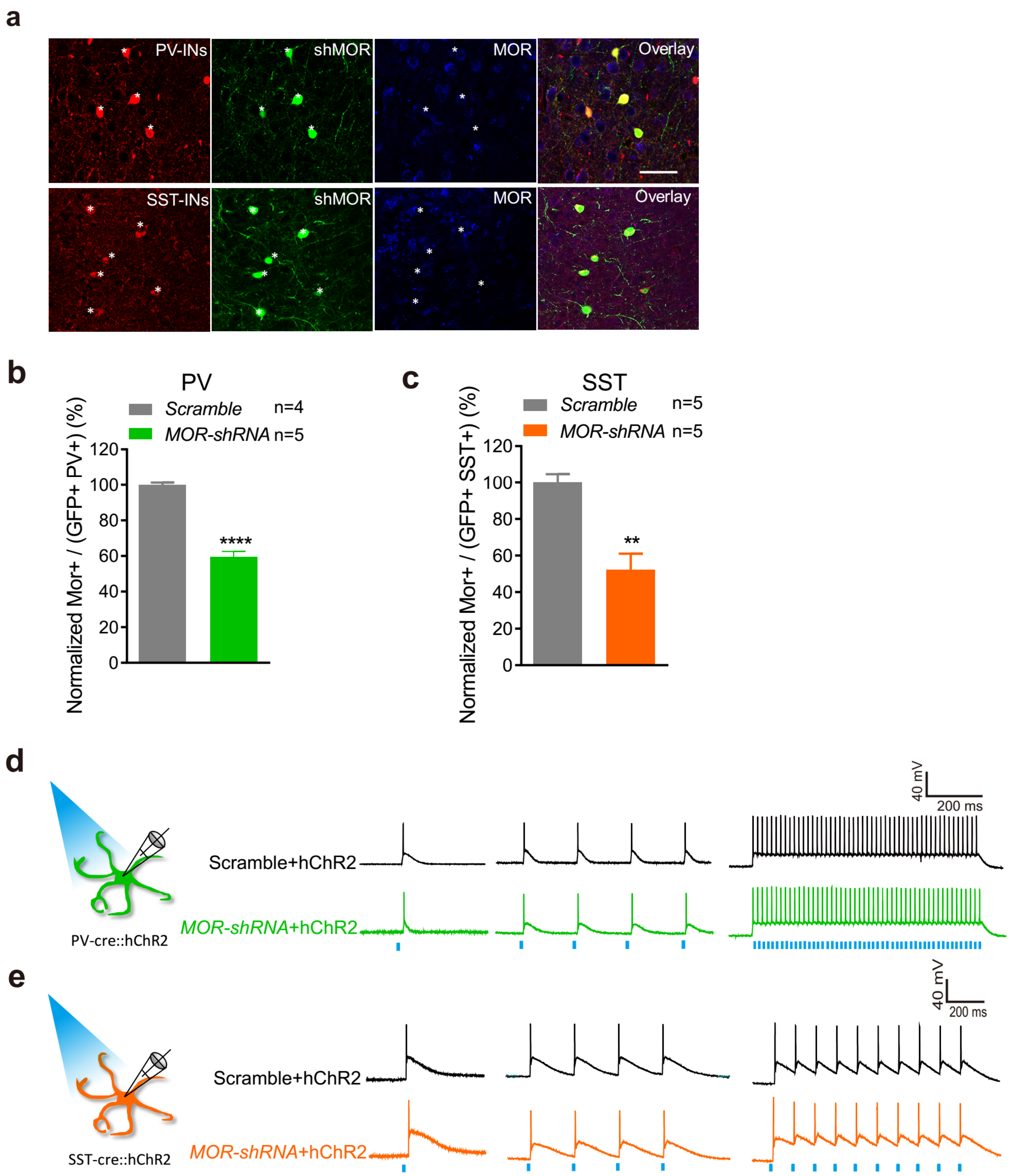

Fig. S2. Knockdown efficiency of *MOR-shRNA* and light-evoked AP firing in SST-INs or PV-INs expressing *Scramble* or *MOR-shRNA*.

a Representative images of SST-INs and PV-INs expressing *Scramble* or *MOR-shRNA* in PrL. Virus infection was monitored by the presence of EGFP. The cell types of neurons and the protein expression of MOR were assessed by specific antibodies, respectively. Scale bar, 20

μm. Green, EGFP; Red, SST/PV; Blue, MOR. White stars indicate the interneurons infected with *MOR-shRNA*. **b** Normalized percentage of MOR^+ cells in $EGFP^+PV^+$-INs (n = 4-5 mice/group; Unpaired Student's *t*-test). **c** Normalized percentage of MOR^+ cells in $EGFP^+SST^+$-INs (n = 5 mice/group; Unpaired Student's *t*-test). **d-e** Diagram and representative recording trace showing the PV-INs (**d**) or SST-INs (**e**) co-expressing hChR2 with *MOR-shRNA* or *Scramble-shRNA* in response to light pulse with different frequency. Data are presented as mean ± S.E.M. $^{**}P < 0.01$, $^{****}P < 0.0001$.

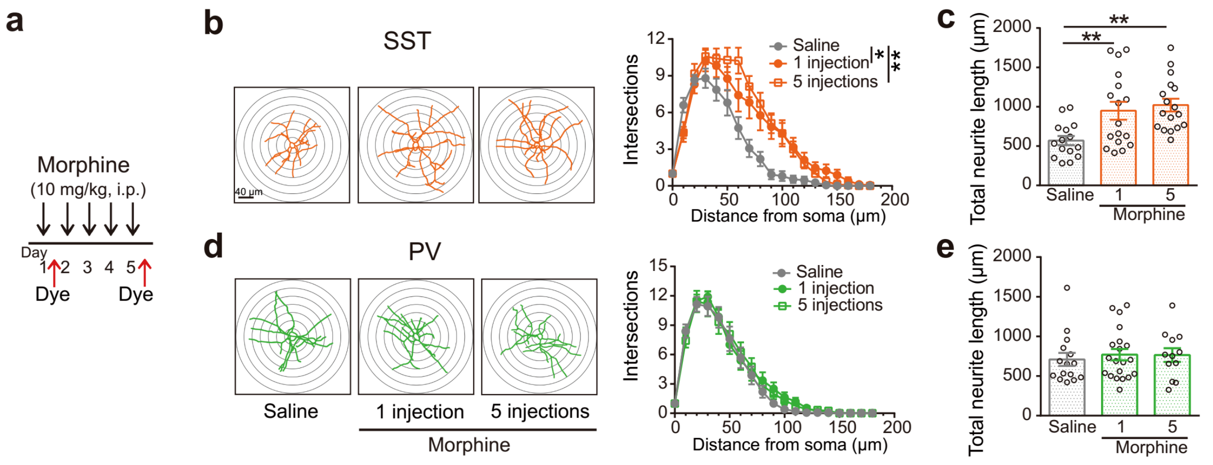

Fig. S3. Morphine increases neurite complexity in SST-INs

a Experimental scheme for morphine and dye injections to study morphological changes of PrL interneurons in *SST-Cre::EYFP* or *PV-Cre::EYFP* mice 12 h after a single or 5 consecutive morphine injections (10 mg/kg, i.p.). **b-e** Sholl analysis (**b, d**) and total neurite length (**c, e**) of SST-INs (**b, c**: n = 15 cells/3 mice in saline group, 17 cells/3 mice in a single or five morphine injections group) and PV-INs (**d, e**: n = 15 cells/3 mice in saline group, 20 or 12 cells/3 mice in a single or five morphine injections group). **b:** Distance from soma: $F_{(18, 828)} = 119.7$, $P < 0.0001$, treatment: $F_{(2, 46)} = 5.242$, $P = 0.0089$, interaction: $F_{(36, 828)} = 4.107$, $P < 0.0001$; **d**: Distance from soma: $F_{(18, 792)} = 197.8$, $P < 0.0001$, treatment: $F_{(2, 44)} = 0.2259$, $P = 0.7987$, interaction: $F_{(36, 792)} = 0.5546$, $P = 0.9848$; Two-way RM ANOVA by Bonferroni's 's *post hoc* test. **c:** Total neurite length: $F_{(2, 46)} = 7.035$, $P = 0.0022$; **e:** Total neurite length: $F_{(2, 44)} = 0.1799$, $P = 0.8360$; One-way ANOVA by Bonferroni's *post hoc* test. Data are presented as mean ± S.E.M. *$P < 0.05$, **$P < 0.01$.

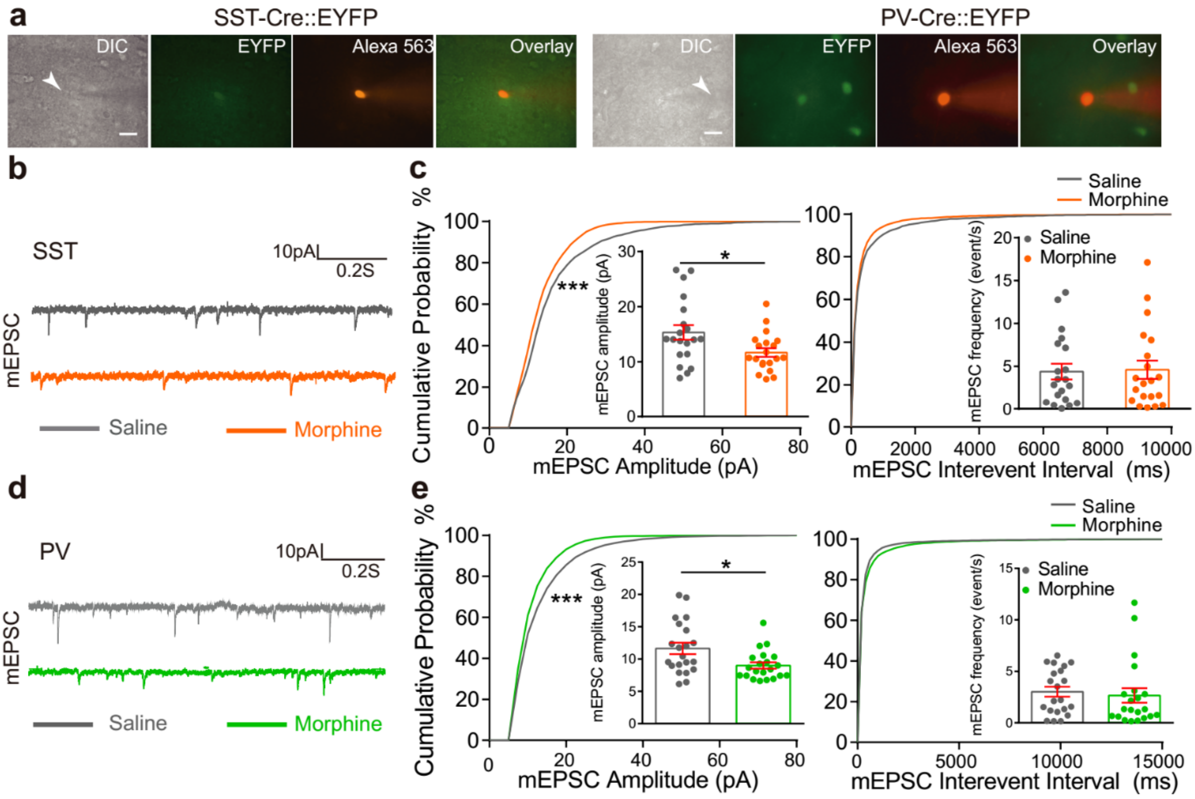

Fig. S4. Morphine decreases the amplitude of mEPSC in SST-INs and PV-INs.

a Representative images of the $EYFP^+$ interneurons in PrL traced in acute slice from *SST-Cre::EYFP* or *PV-Cre::EYFP* mice 12 h after injection of saline or 10 mg/kg morphine. The fluorescent dye tracer was infused after recording. Scale bar: 10 μm. **b** Representative traces of mEPSCs recorded from SST-INs with single saline or morphine exposure in acute slices. **c** The cumulative probability distribution or average amplitude and frequency of mEPSCs recorded from SST-INs (n = 20 cells from 5 mice/group; Mann-Whitney *U* test for amplitude and frequency; Two-sample *Kolmogorov–Smirnov test* for cumulative probability distribution). **d** Representative traces of mEPSCs recorded from PV-INs with single saline or morphine exposure in acute slices. **e** The cumulative probability distribution or average amplitude and frequency of mEPSCs recorded from PV-INs (n = 21 cells/5 mice in each group; Mann-Whitney

U test for amplitude and frequency; Two-sample *Kolmogorov–Smirnov test* for cumulative probability distribution). Data are presented as mean ± S.E.M. *$P < 0.05$, ***$P < 0.001$.

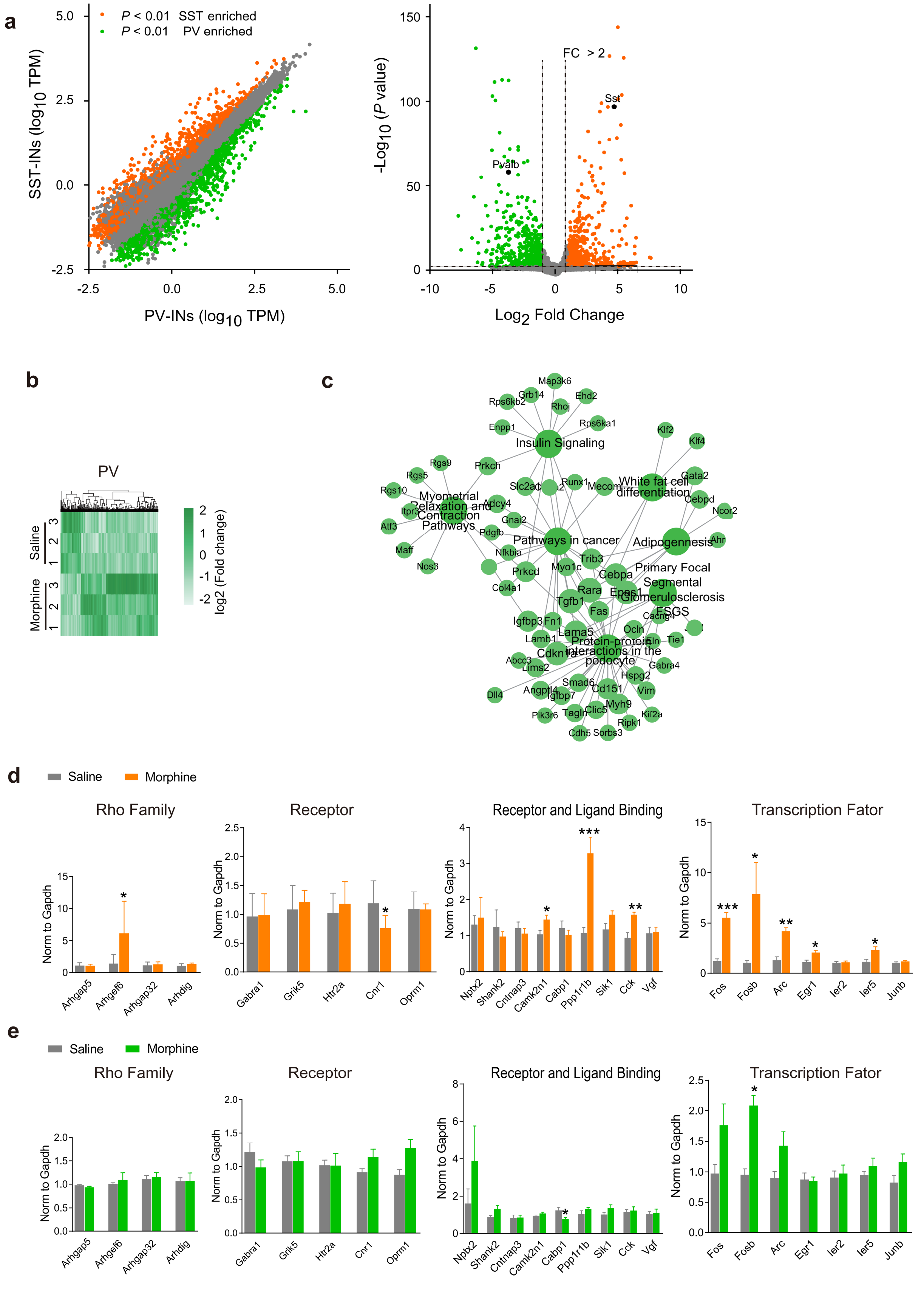

Fig. S5. Expression profiling of the ribosome-associated transcripts reveals the

enrichment of cell type-specific markers and qRT-PCR verification of the morphine-changed transcripts in SST-INs and PV-INs.

a Scatter plot presenting the values of $\log_{10}$ (TPM) for each gene in the SST-INs samples versus PV-INs samples from the PrL of *SST-Cre::RPL22-HA* mice or *PV-Cre::RPL22-HA* mice. Left: Differential enrichment showing 2836 genes (orange dots) enriched in SST-INs and 2789 genes (green dots) enriched in PV-INs with $P < 0.01$. Right: Volcano plot showing ribosome-associated transcripts with at least two-fold enrichment in SST-INs (orange) or PV-INs (green). **b** A heat map of hierarchical clustering of normalized level of ribotag-isolated transcripts in PV-INs (3 mice/group). Each row corresponds to a single gene. **c** Representation of the morphine-regulated signaling network enrichment analysis including all modules and contributing genes in PV-INs. **d-e** qRT-PCR analyzed the ribosome-associated transcripts of genes for Rho family, membrane receptors, transcription factors, ligands and binding proteins in SST-INs (n = 6 mice/group; Unpaired Student's t-test in **d)** and PV-INs (n = 4 mice/group; Unpaired Student's *t*-test in **e**). Data are presented as mean ± S.E.M. *$p < 0.05$, **$P < 0.01$, ***$P < 0.001$.

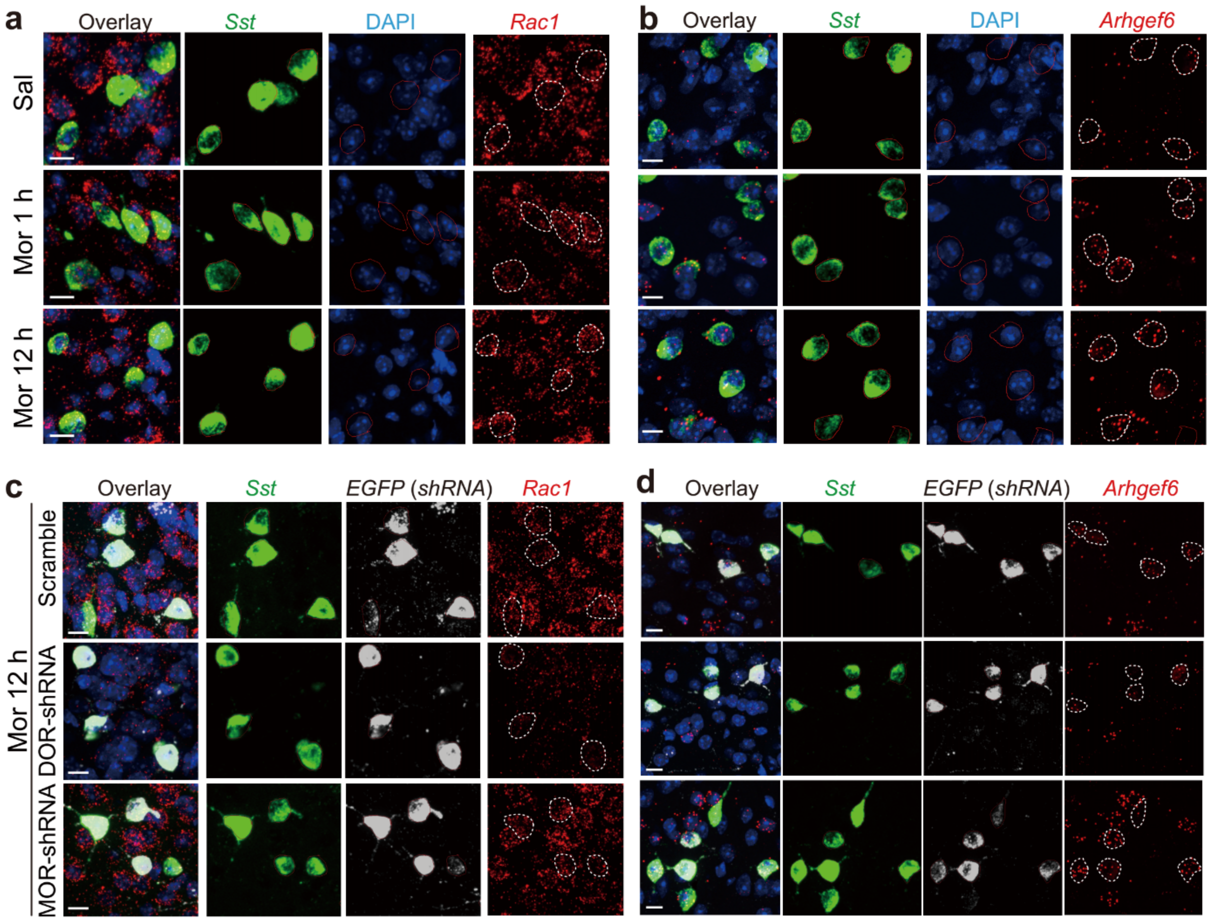

Fig. S6. Representative confocal images showing the distribution of *Rac1* or *Arhgef6* transcripts in SST-INs.

a-b smFISH for *Rac1* or *Arhgef6* transcript in SST-INs 1 h or 12 h after saline or morphine injection (10 mg/kg, i.p.). White dotted circles: SST-INs. **c-d** smFISH for *Rac1* or *Arhgef6* in SST-INs expressing *DOR-shRNA, MOR-shRNA,* or *Scramble-shRNA* 12 h after morphine injection. White dotted circles: SST-INs expressing indicated *shRNA*. Scale bar, 10 µm.

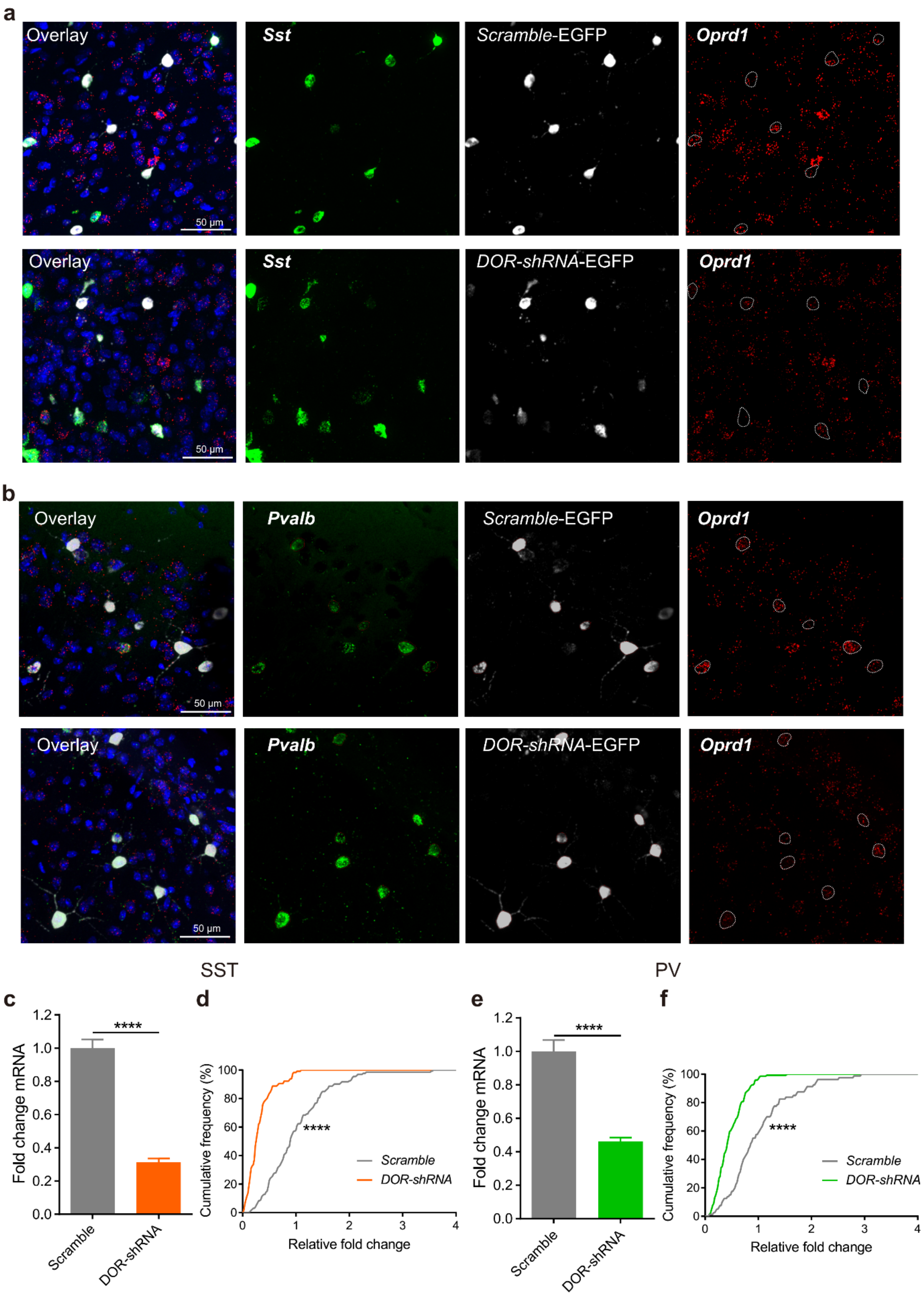

Fig. S7. Verification of the knockdown efficiency of *DOR-shRNA* in SST-INs and PV-INs.

a Representative images of SST-INs expressing *Scramble* or *DOR-shRNA* in PrL. Virus infection was monitored by the probe of *EGFP*. The types of neurons and the expressing of DOR were assessed by specific probe. Green, *Sst*; White, EGFP; Red, *Oprd1*. White dotted lines indicate SST-INs infected with *DOR-shRNA*. **b** Representative images of PV-INs expressing *Scramble* or *DOR-shRNA* in PrL. Virus infection was monitored by the probe of *EGFP*. The types of neurons and the expressing of DOR were assessed by specific probe. Green, *Pvalb*; White, EGFP; Red, *Oprd1*. White dotted lines indicate PV-INs infected with *DOR-shRNA*. **c-d** Quantification of the expression of DOR in SST-INs (n = 133 cells from 3 mice/ in *Scramble* group, n = 116 cells from 3 mice in *DOR-shRNA* group). **e-f** Quantification of the expression of DOR in PV-INs (n = 80 cells/3 mice in *Scramble* group, n = 146 cells/3 mice in *DOR-shRNA* group. Unpaired Student's *t*-test for average fluorescence intensity fold change, and Two-sample *Kolmogorov–Smirnov test* for cumulative probability distribution of fold change). Data are presented as mean ± S.E.M. ****$P < 0.0001$.

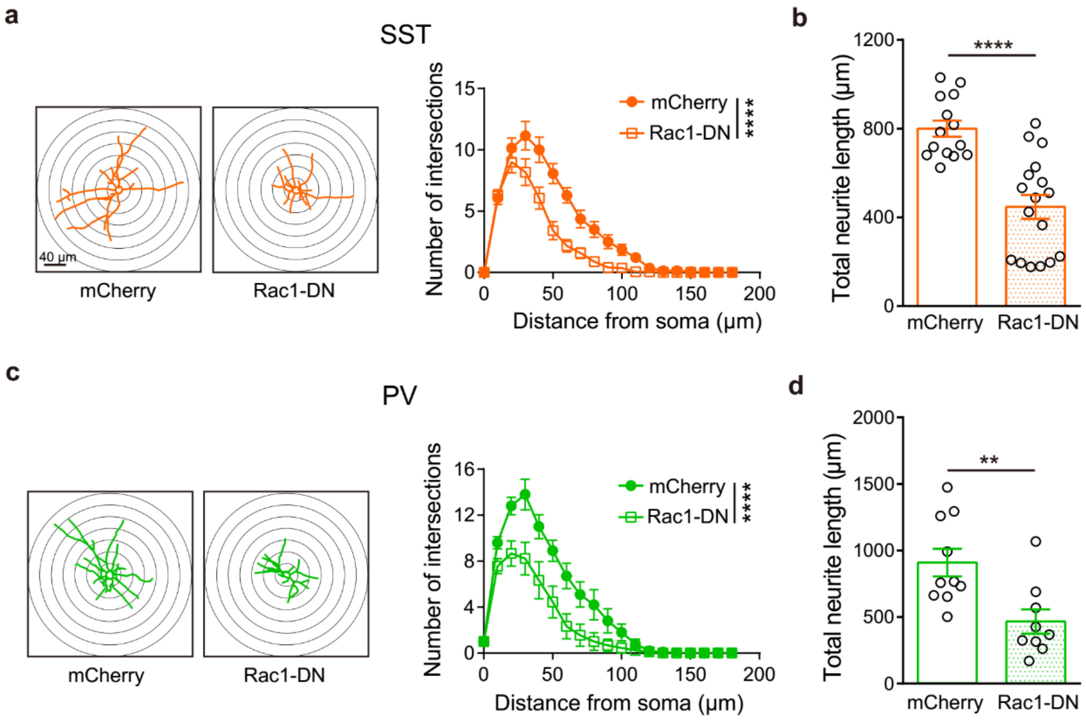

Fig. S8. The effect of Rac1-DN on the morphology of SST-INs and PV-INs.

a Left, representative images of SST-INs expressing mCherry or Rac1-DN after reconstruction and concentric rings for sholl analysis. Right, sholl analysis of SST-INs from saline and morphine treatment mice (n = 14-17 cells/6 mice in each group; Two-way RM ANOVA by Bonferroni's *post-hoc* test. Distance from soma: $F_{(18, 522)} = 104.7$, $P < 0.0001$, virus: $F_{(1, 29)} = 22.18$, $P < 0.0001$, interaction: $F_{(18, 522)} = 5.837$, $P < 0.0001$). **b** Quantification of the total neurite length in SST-INs expressing mCherry or Rac1-DN (Unpaired Student's *t*-test). **c** Left, representative images of PV-INs expressing mCherry or Rac1-DN after reconstruction and concentric rings for sholl analysis. Right, sholl analysis of PV-INs from saline and morphine treatment mice (n = 10-9 cells/ 6 mice in each group; Two-way RM ANOVA by Bonferroni's *post-hoc* test. Distance from soma: $F_{(18, 306)} = 79.56$, $P < 0.0001$, virus: F (1, 17) = 8.74, $P = 0.0088$, interaction: $F_{(18, 306)} = 5.226$, $P < 0.0001$). **d** Quantification of the total neurite length in PV-INs expressing mCherry or Rac1-DN (Unpaired Student's *t*-test). Data are presented as mean ± S.E.M. $^{**}P < 0.01$, $^{****}P < 0.0001$.

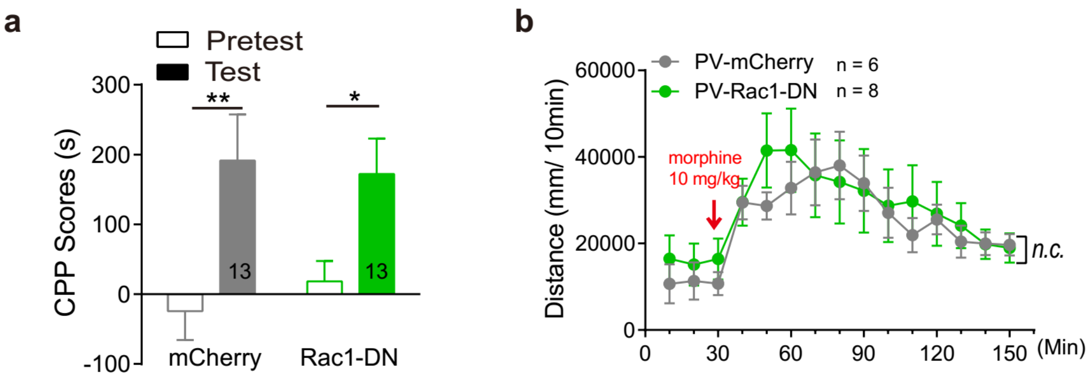

Fig. S9. The effect of expressing Rac1-DN in PV-INs on morphine-induced CPP and hyper-locomotion.

a The effect of down-regulating Rac1 activity of PV-INs on morphine-induced CPP (n = 13 mice/group; Paired Student's *t*-test, Test *vs.* Pre-test, mCherry, P = 0.0054, Rac1-DN, P = 0.0240). **b** The effect of down-regulating Rac1 activity of PV-INs on morphine-induced hyper-locomotion (n = 6 mice in mCherry group, n = 8 mice in Rac1-DN group; Two-way RM ANOVA by Bonferroni's *post-hoc* test, time: $F_{(11, 132)} = 6.156$, $P < 0.0001$, virus: $F_{(1, 12)} = 0.07781$, $P = 0.7850$, interaction: $F_{(11, 132)} = 0.8897$, $P = 0.5522$). Data are presented as mean ± S.E.M. *$P < 0.05$, **$P < 0.01$ *vs* control virus group.

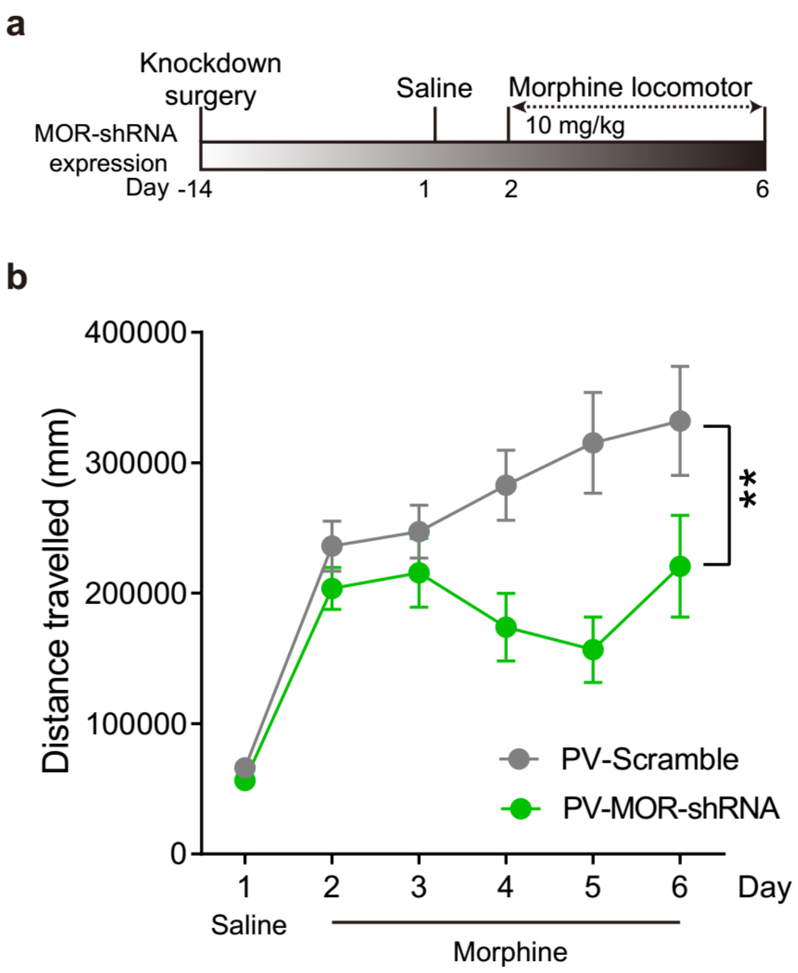

Fig. S10. The effect of knockdown of MOR in PV-INs on morphine-induced behavioral sensitization.

a Experimental schedule for morphine-induced behavioral sensitization. **b** Decrease in locomotor response to morphine was seen in mice expressing *MOR-shRNA* in PV-INs in PrL (n = 11 mice in *Scramble* group, n = 13 mice in *MOR-shRNA* group; Two-way RM ANOVA by Bonferroni's *post-hoc* test, day: $F_{(5, 110)} = 23.33$, $P < 0.0001$, virus: $F_{(1, 22)} = 9.264$, $P = 0.0060$, interaction: $F_{(5, 110)} = 3.614$, $P = 0.0046$). Data are presented as mean ± S.E.M. $**P < 0.001$ compared with *Scramble*.

Table S1. Intrinsic electrophysiological properties of SST-INs and PV-INs in prelimbic cortex.

	Treatment	Rin (MΩ)	Threshold (mV)	Amplitude (mV)	Half-width (ms)	AHP (mV)
SST-INs	Saline (n = 35)	348.0 ± 24.5	-37.79 ± 1.34	73.78 ± 1.48	0.90 ± 0.041	16.22 ± 0.64
	Morphine (n = 34)	366.5 ± 21.8	-37.82 ± 0.97	70.69 ± 1.63	0.84 ± 0.040	16.83 ± 0.68
PV-INs	Saline (n = 29)	261.0 ± 15.4	-32.96 ± 1.11	56.03 ± 1.74	0.59 ± 0.026	22.37 ± 0.96
	Morphine (n = 27)	258.4 ± 14.0	-33.35 ± 1.07	56.60 ± 1.51	0.57 ± 0.024	22.46 ± 0.76

(Unpaired Student's t-test, $P > 0.05$). Saline treatment groups were compared with morphine treatment groups in SST-INs or PV-INs. Data are presented as mean ± S.E.M.

Table S2. DNA Sequence of qRT-PCR Primer			
**Gene**	**Sequence (5'-3')**	**Amplicon Size**	**Primer Bank ID**
GAPDH-F	TGTGTCCGTCGTGGATCTGA	77	homemade
GAPDH-R	CCTGCTTCACCACCTTCTTGA		
Arhgap5-F	TTGGACTCTCTGGGACTGAAA	118	6753112a1
Arhgap5-R	AGCACAGAAGTATGCTCTGGA		
Arhgef6-F	AATCCAGAAGAACGCCTTGTG	117	22779885a1
Arhgef6-R	CACTACCCCATTTTTCAGCGA		
Arhgap32-F	ATGAAGTCTCGCCCAACAAAA	103	28893539a1
Arhgap32-R	CAAACCCAGAATTGAGGAGGTG		
Arhgdig-F	GTCAACTCCATCAGATGAGGTG	160	6679989a1
Arhgdig-R	GGGGTCCATAATGGGTGGC		
Gabra1-F	AAAAGTCGGGGTCTCTCTGAC	138	6753936a1
Gabra1-R	CAGTCGGTCCAAAATTCTTGTGA		
Grik5-F	ATAGTCGCCTTCGCCAATCC	215	6680093a1
Grik5-R	GTGTCCGTGGTCTCGTACTG		
Htr2a-F	TAATGCAATTAGGTGACGACTCG	119	27753985a1
Htr2a-R	GCAGGAGAGGTTGGTTCTGTTT		
CnR1-F	TCTTGTGAGAATTGGTTGGCAA	250	14161704a1
CnR1-R	CATCTCCATAGGTCCCCTTATCA		

OPRM1-F	TCCGACTCATGTTGAAAAACCC	96	17046157a1
OPRM1-R	CCTTCCCCGGATTCCTGTCT		
Nptx2-F	TCAAGGACCGCTTGGAGAG	176	7949098a1
Nptx2-R	CGAGGTCTCATTATGAAGCAGG		
Shank2-F	AGAGGCCCCAGCTTATTCCAA	224	28804747a1
Shank2-R	CAGGGGTATAGCTTCCAAGGC		
Cntnap3-F	CCCCTTAACCTGGAACCCTAA	135	124487466c3
Cntnap3-R	GGTCTTATGGCTCCGTTATCAAA		
Camk2n1-F	CTTGAACCGCCAAAAGGCATT	139	26356604a1
Camk2n1-R	CAAATACCATGCAAAACAGGCA		
Cabp1-F	CTCTCAAGAAAGATGCGCCAG	132	7304939a1
Cabp1-R	GTGCATGACCGCACAGTTC		
Ppp1r1b-F	CCAACCCCTGCCATGCTTT	113	21536256a1
Ppp1r1b-R	TTGGGTCTCTTCGACTTTGGG		
Sik1-all-F	TCATGTCGGAGTTCAGTGCG	151	6754746a1
Sik1-all-R	ACCTGCGTTTTGGTGACTCG		
CCK-F	AAGAGCGGCGTATGTCTGTG	161	291575144c1
CCK-R	CATCCAGCCCATGTAGTCCC		
Vgf-F	TTCAGTCCGAGCAATGCTAAG	195	86476053c3
Vgf-R	AGCCTGGAATTGGGAAGGGA		
Fos-F	CGGGTTTCAACGCCGACTA	166	6753894a1

Fos-R	TTGGCACTAGAGACGGACAGA		
Fosb-F	TTTTCCCGGAGACTACGACTC	174	6679827a1
Fosb-R	GTGATTGCGGTGACCGTTG		
Arc-F	AAGTGCCGAGCTGAGATGC	105	9055166a1
Arc-R	CGACCTGTGCAACCCTTTC		
Egr1-F	CAGTCCCATCTACTCGGCTG	180	76559936c3
Egr1-R	TGTGGAAACAGATAGTCAGGGAT		
Ier2-all-F	TGACTCTGTCGGTATGGAAGAT	124	31542990a1
Ier2-all-R	ACCTTGGCTGAGAGGTAGACC		
Ier5-F	TCACCGCATCGTCAGCATC	131	6754284a1
Ier5-R	GGTCACTCAGGTAGACTTGGC		
Junb-F	TCACGACGACTCTTACGCAG	125	6680512a1
Junb-R	CCTTGAGACCCCGATAGGGA		
